# Regulation of the Nrf2/HO-1 axis by mesenchymal stem cells-derived extracellular vesicles: implications for disease treatment

**DOI:** 10.3389/fcell.2024.1397954

**Published:** 2024-06-10

**Authors:** Hua Su, Zhongan Wang, Lidan Zhou, Dezhi Liu, Nian Zhang

**Affiliations:** ^1^ Xingyi People’s Hospital, Xingyi, China; ^2^ The Fifth Affiliated Hospital of Zhengzhou University, Zhengzhou, China

**Keywords:** mesenchymal stem cell, extracellular vesicle, exosome, Nrf2, HO-1

## Abstract

This comprehensive review inspects the therapeutic potential of mesenchymal stem cell-derived extracellular vesicles (MSC-EVs) across multiple organ systems. Examining their impact on the integumentary, respiratory, cardiovascular, urinary, and skeletal systems, the study highlights the versatility of MSC-EVs in addressing diverse medical conditions. Key pathways, such as Nrf2/HO-1, consistently emerge as central mediators of their antioxidative and anti-inflammatory effects. From expediting diabetic wound healing to mitigating oxidative stress-induced skin injuries, alleviating acute lung injuries, and even offering solutions for conditions like myocardial infarction and renal ischemia-reperfusion injury, MSC-EVs demonstrate promising therapeutic efficacy. Their adaptability to different administration routes and identifying specific factors opens avenues for innovative regenerative strategies. This review positions MSC-EVs as promising candidates for future clinical applications, providing a comprehensive overview of their potential impact on regenerative medicine.

## Graphical Abstract

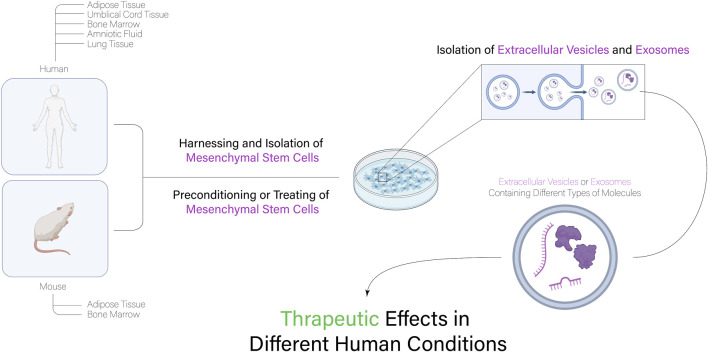

## 1 Introduction

Mesenchymal stem cells (MSCs) are adult stem cells sourced from bone marrow and adipose tissue. They are characterized by their capacity for self-renewal and differentiation into diverse cell types. Their distinct attributes make them valuable in combating various human diseases, such as cardiovascular events (Guo et al., 2020), neurological disorders (Andrzejewska et al., 2021), inflammatory conditions (Shi et al., 2018), and liver diseases (Wu and Meng, 2021). MSCs exhibit a “homing” ability, migrating to inflammation or tumor sites. These features find applications in regenerative medicine, facilitating tissue repair and immunomodulation for treating autoimmune diseases and preventing graft-versus-host disease (Wu and Meng, 2021). In many of these diseases, treatment with MSCs has shown promising results at the clinical level (Rodríguez-Fuentes et al., 2021).

The Nrf2/HO-1 pathway is a vital cellular defense mechanism that orchestrates the response to oxidative stress and inflammation, exerting profound effects on cellular homeostasis and disease pathology. At the core of this pathway is nuclear factor erythroid 2-related factor 2 (Nrf2), a transcription factor that regulates the expression of genes encoding antioxidant and detoxification enzymes. In the presence of oxidative stress or other insults, reactive oxygen species (ROS) modify critical cysteine residues on Keap1, disrupting its interaction with Nrf2 and allowing Nrf2 to evade degradation. This results in the translocation of Nrf2 into the nucleus, where it forms heterodimers with small Maf proteins and binds to antioxidant response elements (AREs) in the promoter regions of target genes, thereby activating their transcription. HO-1 is an inducible stress-response protein that catalyzes the degradation of heme into biliverdin, carbon monoxide (CO), and free iron. This enzymatic activity not only serves to detoxify heme, a pro-oxidant molecule released during oxidative stress and inflammation, but also generates bioactive metabolites with potent anti-inflammatory, antioxidative, and cytoprotective properties. The importance of the Nrf2/HO-1 pathway in maintaining cellular redox balance and combating oxidative stress-related damage is underscored by its involvement in various pathological processes. Dysregulation of this pathway has been implicated in the pathogenesis of numerous diseases, including atherosclerosis, neurodegenerative disorders, cancer, and metabolic syndrome (Zhang et al., 2021; Mansouri et al., 2022).

Extracellular vesicles (EVs) are a range of membrane-enclosed vesicles encompassing exosomes, apoptotic bodies, and autophagic vesicles. A variety of cells can secrete the EVs and exosomes. These EVs and exosomes originating from MSCs are tiny vesicles packed with bioactive substances, including proteins and nucleic acids. These vesicles could possess potent anti-inflammatory properties and the ability to modulate the immune system, making them highly promising for addressing inflammatory and autoimmune disorders. Moreover, they play a crucial role in tissue regeneration by stimulating cell growth and angiogenesis. Ongoing clinical trials explore the application of MSC-derived microvesicles in conditions like graft-versus-host disease, chronic kidney disease, and neurological ailments. Their use in personalized medicine is also under investigation, presenting a cell-free alternative to MSC-based therapies (Nikfarjam et al., 2020; Liu et al., 2024).

In our comprehensive review, we meticulously examined the pivotal role of exosomes derived from MSCs in modulating the Nrf2/HO-1 axis within human pathological conditions. A comprehensive search on PubMed was conducted using the keywords (Exosome OR Extracellular Vesicle) AND (Mesenchymal Stem Cell) AND (NRF2 OR HO-1) spanning from January 2011 to January 2024 to identify relevant articles on this topic. Inclusion criteria comprised studies focusing on the role of NRF2/HO-1 overexpressing exosomes derived from mesenchymal stem cells in the treatment of various human diseases. Exclusion criteria encompassed case reports, review articles, editorials, abstracts, and bioinformatic studies. Our analysis delved into the intricate mechanisms underlying the therapeutic effects of MSC-derived exosomes, shedding light on their potential as therapeutic agents.

## 2 Nrf2/HO-1 axis regulation

**FIGURE 1:**
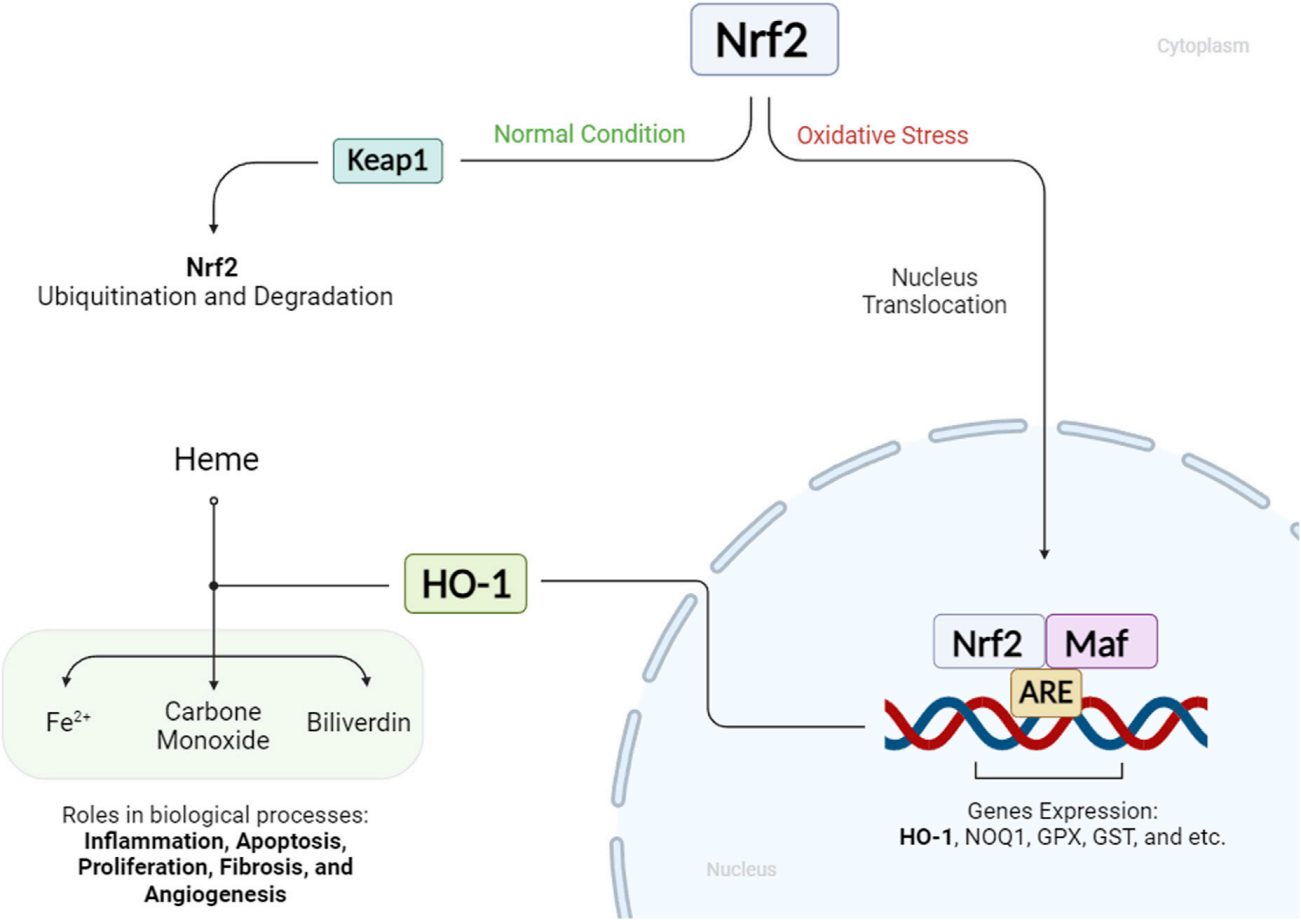
Status of HO-1 and Nrf2 in cellular conditions: When a cell is under oxidative stress, Nrf2 enhances HO-1 expression. HO-1 mediates the production of Fe^2+^, carbon monoxide, and biliverdin from heme molecules. These HO-1 byproducts play key roles in various biological processes.

The Nrf2/HO-1 axis is a crucial regulatory pathway involving the transcription factor Nrf2 and the inducible enzyme HO-1. Under normal conditions, Nrf2 is controlled by Kelch-like ECH-associated protein 1 (Keap1), which promotes its ubiquitination and degradation. However, stimuli such as electrophiles, reactive oxygen species, and phosphorylation events can lead to Nrf2 stabilization and its translocation into the nucleus. In the nucleus, Nrf2 dimerizes with tiny Maf proteins and binds to antioxidant response elements (AREs), activating the transcription of cytoprotective genes, including HO-1. HO-1, in turn, catalyzes the breakdown of heme into iron ions, biliverdin, and carbon monoxide, with biliverdin further converted to bilirubin. These by-products play vital roles in biological processes such as inflammation, apoptosis, proliferation, fibrosis, and angiogenesis (Figure 1) (Liu C. et al., 2023; Ghareghomi et al., 2023). Although HO-1 is consistently present in specific tissues under normal conditions, its increased expression in the face of stress, inflammation, and diseases implies a protective function. The products generated by HO-1 exhibit antioxidant, anti-inflammatory, and cytoprotective properties. Elevated levels of HO-1 have been associated with non-malignant disease reversal, as it helps alleviate oxidative stress, modulate signaling pathways, and control the expression of genes (Yang and Wang, 2022). HO-1 has garnered increased attention due to its multifaceted role in cellular processes, particularly its capacity to regulate cell death pathways such as ferroptosis. Beyond its antioxidant properties, HO-1 exhibits diverse biological functions, influencing inflammation, apoptosis, and cell survival. Its implication in a broad spectrum of diseases, including neurodegenerative and cardiovascular diseases, positions HO-1 as a potential therapeutic target. Notably, its involvement in regulating ferroptosis, a form of iron-dependent cell death, adds significance to its study, as ferroptosis is implicated in various diseases (Kajarabille and Latunde-Dada, 2019; Tang et al., 2021).

The upstream regulation of HO-1 is primarily achieved through Nrf2 in MSCs. Nrf2, a transcription factor responding to oxidative stress, induces various antioxidants, including superoxide dismutase and HO-1, in MSCs, enhancing their anti-oxidative and anti-apoptotic capabilities. Thus, Nrf2-mediated induction of antioxidants, particularly HO-1, plays a crucial role in promoting the survival and resistance of MSCs to cytotoxic conditions (Mohammadzadeh et al., 2012). HO-1 undergoes precise transcriptional regulation by redox-sensitive transcription factors such as AP-1, NF-κB, HIF, and Nrf2, responding to various stressors. These factors activate HO-1 expression in the face of oxidative, electrophilic, hypoxic, and inflammatory challenges. The downstream impact of HO-1 is diverse, encompassing anti-inflammatory, antioxidant, and anti-apoptotic functions. Its byproducts, biliverdin, and carbon monoxide, act as potent antioxidants, and controlling iron metabolism contributes to cellular redox balance. Nevertheless, the consequences of HO-1 activation are dual, manifesting both protective and potentially harmful effects, dependent on the disease (malignant and non-malignant) (Loboda et al., 2016). The crosstalk between nuclear heme oxygenase-1 (HO-1) and various signaling pathways highlights its significant role in cellular responses to stress and disease conditions. Nuclear HO-1, beyond its enzymatic activity, interacts with key transcription factors such as Nrf2, Bach1, NF-κB, and STAT3, influencing their transcriptional activities and thereby modulating the expression of genes critical for antioxidant responses, inflammation, and cell proliferation. In the context of oxidative stress, nuclear HO-1 enhances the stabilization and activity of Nrf2, which in turn induces the expression of antioxidative genes such as NQO1 and G6PDH. Meanwhile, competing interactions with Bach1 downregulate HO-1, thereby influencing tumor metastasis and growth. Additionally, the interaction of HO-1 with the MEK/ERK pathway suggests a role in neuroprotection and cellular proliferation. In cancer, nuclear HO-1 affects the JAK-STAT3 pathway by modulating STAT3 activity, impacting tumor growth and survival pathways. The involvement of HO-1 with NF-κB indicates its role in inflammation and apoptosis, underpinning its potential in therapeutic strategies against diseases like cancer and inflammation-related disorders. This complex network of interactions emphasizes the multifaceted role of nuclear HO-1 in cellular defense mechanisms (Yang and Wang, 2022). Many other downstream signaling pathways, such as Wnt (Vanella et al., 2013), JNK (Yin et al., 2020), and NF-κB (Ning et al., 2021), are also affected by HO-1 upregulation, leading to cell proliferation and preventing cell death (Kim et al., 2016).

Nrf2 is also under the control of many other regulators, such as Akt (Chen et al., 2022), Sirtuins (Pan et al., 2016), IL-10 (Su et al., 2023), and MAPK signaling (Sun et al., 2009), all safeguarding cells from oxidative stress. Under normal conditions, Nrf2 is tightly regulated by the Keap1-CUL3-RBX1 E3 ubiquitin ligase complex, leading to Nrf2 degradation via the ubiquitin-proteasome pathway. Keap1 acts as a sensor for oxidative stress, binding to Nrf2 and promoting its ubiquitination. This constitutive degradation mechanism maintains low levels of Nrf2 in the cell. However, during oxidative stress or exposure to electrophiles, chemical inducers disrupt the Keap1-Nrf2 interaction by reacting with cysteine residues in Keap1. This disruption prevents Nrf2 ubiquitination, allowing Nrf2 to accumulate, translocate to the nucleus, and initiate the transcription of genes involved in the antioxidant response. This way, Nrf2 is activated in response to cellular stress, ensuring a controlled and rapid defense against oxidative damage (Baird and Yamamoto, 2020).

**FIGURE 2:**
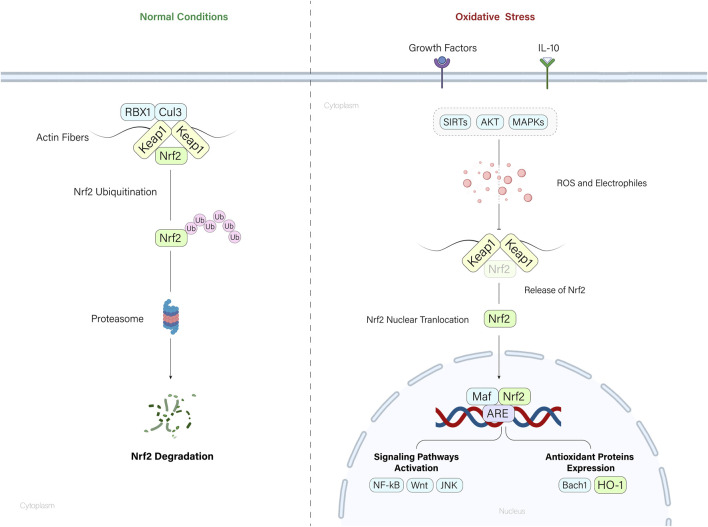
Nrf2/HO-1 axis regulation. Under normal conditions, the Keap1-mediated ubiquitination of Nrf2 molecules degrades the Nrf2 molecules by cytoplasmic proteasomes. When cells encounter oxidative stress, accumulation of ROS and electrophiles leads to the Nrf2 release from the Nrf2-Keap1 complex. Released Nrf2 molecules translocate to the nucleus and mediate the expression of key regulatory molecules and pathways within the cell.

During oxidative stress conditions, damaged somatic cells release respiring mitochondria, triggering MSCs to activate their anti-apoptotic response. MSCs uptake these mitochondria through autophagy, leading to the induction of the cytoprotective enzyme HO-1 (Luo et al., 2023). This, in turn, stimulates mitochondrial biogenesis in MSCs, enhancing their ability to donate healthy mitochondria to distressed cells. This mitochondria-mediated communication is crucial for MSCs to rescue cells from apoptosis, suggesting the potential of targeting the mitochondria-HO-1 axis for improving MSC-based therapies (Figure 2) (Mahrouf-Yorgov et al., 2017).

## 3 Nrf2/HO-1 regulation in human diseases

Table 1 summarizes key information on the treated medical conditions using MSC-derived EVs. It includes details on the condition addressed, the source of exosomes, the regulatory axis involved, the experimental models employed, and the observed outcomes.

**TABLE 1:** Summary of MSC-EVs in human diseases.

Study	Condition	EVs origin (preconditioning)	Regulatory axis	Experiment models	Outcomes	Treatment dose	Treatment duration
Nervous System							
Zhang et al. (2024a)	TBI	Human umbilical cord MSCs	lncRNA TUBB6/Nrf2	*In vivo* (mouse TBI model)	↓ Neural inflammation	200 μg/mL	NM
				*In vitro* (mouse cortical neurons)	↓ Neuron cell death		
				*In vitro* (HucMSCs)	↓ Inflammation		
					↓ Ferroptosis		
Liu et al. (2021a)	SCI	Melatonin-treated MSCs	USP29 ↑/Nrf2 ↑/HO-1 ↑	*In vivo* (mouse SCI model)	↑ Motor recovery	4 mg/mL	24 h
				*In vitro* (BV2 microglia, RAW264.7 macrophages)	↑ Functional recovery		
				*In vitro* (BMSCs pretreated with melatonin)			
Liu et al. (2021b)	Inflammation injury	IL-1-treated MSCs	Nrf2 ↑/HO-1 ↑	*In vivo* (mouse SE model)	↓ Inflammatory responses	10 ng/mL	24 h
				*In vitro* (LPS-induced astrocytes)	↓ Cognitive impairment		
Liu et al. (2020)	Hypoxia-reperfusion Injury	hNSCs	Nrf2 ↑	*In vitro* (hNSCs)	↓ Neuronal apoptosis	50 μg/mL	24 h
				*In vitro* (neurons)	↑ Elongation of neuronal axons		
					↑ Angiogenesis		
Guo et al. (2022)	Hypoxia-reperfusion Injury	MSCs	Nrf2 ↓	*In vitro* (hippocampal neurons)	↓ Neuronal apoptosis	20 μg/mL	12 h
				*In vitro* (MSCs)	↓ Oxidative stress		
Huang et al. (2022)	Methotrexate-induced Injury	Adipose MSCs	Nrf2 ↑/ARE ↑	*In vitro* (Hippocampal neurons)	↓ Oxidative stress	10 or 20 ug/mL	24 h
				*In vivo* (SD rats)			
Wang et al. (2023)	VaD	Human umbilical cord MSCs	PI3K/AKT ↑/Nrf2 ↑	*In vivo* (VaD rat model)	↓ Neurological impairment	NM	7 days
				*In vitro* (HucMSCs)	↓ Microglial M1 polarization		
					↓ Oxidative stress		
Provenzano et al. (2022)	ALS	Bone marrow MSCs	Nrf2 ↑	*In vivo* (VaD rat model)	↓ Mouse and human ALS astrocytes’ neurotoxicity towards motor neurons	NM	24 h and 48h
				*In vitro* (Mouse Astrocyte)	↓ Neuroinflammation		
				*In vitro* (Human Astrocyte)	↓ Oxidative stress		
				*In vitro* (MSCs)			
Hu et al. (2023)	ICH	Bone marrow MSCs (miR-23b)	PTEN ↓/Nrf2 ↓	*In vivo* (ICH rat model)	↓ Oxidative stress	100 μg/mL	1 day
				*In vitro* (microglia/macrophage)	↓ Pyroptosis		
				*In vitro* (hippocampal neuron)	↓ Brain edema after ICH		
				*In vitro* (MSCs)	↑ Behavioral functions		
Liu et al. (2022)	dNCR	Bone marrow MSCs	SIRT1 ↑/Nrf2 ↑/HO-1 ↑	*In vitro* (MSCs)	↓ Cognitive impairment	50 μg/mouse	24-h
				*In vivo* (dNCR mouse model)	↓ Ferroptosis		
				*In vitro* (hippocampal neuron)			
Luo et al. (2021)	Seizure	Human umbilical cord MSCs	Nrf2 ↑	*In vitro* (hippocampal neuron)	↓ Oxidative stress	5–30 μg/mL	24 h
				*In vitro* (HucMSCs)	↓ Mitochondrial dysfunction		
				*In vivo* (Seizure mouse model)	↓ Sequelae of seizures		
Integumentary System							
Cheng et al. (2024)	Diabetic Wound	Bone marrow MSCs (HO-1)	HO-1 ↑	*In vitro* (MSCs)	↑ Proliferation and migration of fibroblasts	0.2 mL	24–72 h
				*In vitro* (HUVECs)	↑ Proliferation and migration of keratinocytes		
				*In vitro* (Fibroblasts)	↑ Migration and angiogenesis activity of HUVECs		
				*In vitro* (Keratinocytes)	↑ Cutaneous wound healing		
				*In vivo* (Diabetic mouse model)	↑ re-epithelialization and vascularization in the wound sites		
Chen et al. (2023)	Diabetic Wound	Bone marrow MSCs (circ-ITCH)	TAF15 ↑/Nrf2 ↑	*In vitro* (MSCs)	↑ Angiogenesis ability of HUVECs	10 mg/kg	NM
				*In vitro* (HUVECs)	↓ Ferroptosis of HUVECs		
				*In vivo* (Diabetic mouse model)	↑ Wound healing in mice		
Wang et al. (2022)	Diabetic Wound	Bone marrow MSCs	-	*In vitro* (MSCs)	↑ Wound closure	100 μg/mL	Days 1 and 7
				*In vitro* (EPCs)	↑ Re-epithelization		
				*In vivo* (Diabetic mouse model)	↑ Collagen deposition		
					↑ Neovascularization		
Wang et al. (2020)	Oxidative Stress-induced Skin Injury	Human umbilical cord MSCs	Nrf2 ↑	*In vitro* (Keratinocytes)	↓ Oxidative injury	0.02 μg/μL	12h
					↑ Antioxidant activity		
					↑ Oxidative responsiveness		
				*In vitro* (HucMSCs)	↓ UV-induced histological injury		
				*In vivo* (Mice: UV-induced skin injury)	↓ Inflammatory responses		
					↓ Collagen deposition		
Respiratory System							
Zhang et al. (2023)	CPB-related Acute Lung Injury	Bone marrow MSCs	Akt ↑/Nrf2 ↑/HO-1 ↑	*In vitro* (MSCs)	↓ Inflammatory responses	100 µg/rat	48h
				*In vivo* (CPB mouse model)	↓ Oxidative stress		
					↓ Histological changes		
					↓ ROS production		
Gong et al. (2023)	SM-associated acute lung injury	Human umbilical cord MSCs	CAV1 ↓/Nrf2 ↑/HO-1 ↑	*In vitro* (BEAS-2B cells)	Pneumonocyte oxidative stress and apoptosis	20 mg/mL	Days 1 and 3
				*In vitro* (Human lung fibroblasts)	Activities of antioxidant enzymes		
				*In vivo* (Acute lung injury mouse model)			
Xu et al. (2022a)	Acute Lung Injury	Human amniotic MSCs (Nrf2)	Nrf2 ↑/HO-1 ↑	*In vitro* (hAMSCs)	↓ Lung injury in LPS-challenged mice	200 µL	2 and 12 h
				*In vivo* (ALI mouse model)	↓ Apoptosis		
					↓ Infiltration of neutrophils and macrophages		
					↓ Pro-inflammatory cytokine expression		
					↓ NLRP3 inflammasome		
					↑ Polarization of M2 macrophages		
Zhao et al. (2022a)	Acute Lung Injury	Human umbilical cord MSCs	Nrf2 ↑/HO-1 ↑	*In vitro* (HucMSCs)	↑ Polarization of M2 macrophages	50 μg	24 h
				*In vivo* (ALI mouse model)	↑ Tissue repair		
					↑ Inflammatory mediators		
					↑ Oxidative mediators		
Li et al. (2023a)	Sepsis-Associated ARDS	Bone marrow MSCs	Nrf2 ↑	*In vitro* (MSCs)	↓ Mitochondrial Dysfunction	100 μg/mL	48 h
				*In vitro* (AECII)	↓ Sepsis-Treated AECII Apoptosis		
					↓ Sepsis-Induced ARDS		
					↓ Sepsis-Induced Inflammatory Cytokine Release		
Dong et al. (2022)	Hyperoxia-induced multi-organ injury	Human umbilical cord MSCs	HO-1 ↑	*In vitro* (HucMSCs)	↑ Survival rate	40 μL	Day 7
				*In vitro* (lung, heart, and kidney tissues)	↑ Lung development		
					↓ Hyperoxia-induced lung inflammation		
					↓ Hyperoxia-induced oxidative stress		
Zulueta et al. (2018)	CF	Human lung MSCs	PPARγ ↑/HO-1 ↑	*In vitro* (Cellular model of CF)	↓ Inflammatory cytokines	30 μL	30h
					↑ Anti-oxidant intrinsic defenses		
Digestive System							
Zuo et al. (2023)	Ischemia-reperfusion liver injury	Bone marrow MSCs (HO-1)	miR-214-3p ↑/COX-2 ↑	*In vivo* (liver transplantation mouse model)	↓ Ferroptosis	20 μM	24 h
				*In vitro* (MSCs)			
Li et al. (2022a)	Ischemia-reperfusion liver injury	Bone marrow MSCs (HO-1)	miR-29a-3p	*In vivo* (Steatotic Liver IRI mouse model)	↓ Ferroptosis	20 μM	24 h
				*In vitro* (MSCs)			
Wu et al. (2022)	Ischemia-reperfusion liver injury	Bone marrow MSCs (HO-1)	miR-124-3p	*In vitro* (Steatotic hepatocytes)	↓ Ferroptosis	2.5 × 1,010 particles in 500 μL of PBS	24 h
Sun et al. (2017)	Ischemia-reperfusion liver injury	Adipose MSCs	HO-1 ↑	*In vitro* (MSCs)	↓ Inflammation	100 μg per rat	Each 1, 12, and 36 h
				*In vitro* (PBMCs)	↓ Apoptosis		
					↓ Oxidative stress		
Zhao et al. (2022b)	Acute Liver Injury	Bone marrow MSCs (Baicalin)	Keap1 ↑/Nrf2 ↑	*In vitro* (MSCs)	↓ Inflammation	150 μg	Single dose
				*In vitro* (Hepatocyte)	↓ Ferroptosis		
Liu et al. (2023b)	Liver Fibrosis	MSCs	Nrf2 ↑/HO-1 ↑	*In vitro* (MSCs)	↓ Inflammatory responses	10^8^ particles/mL	24 h
				*In vitro* (LX-2, RAW264.7 macrophages)	↓ CCl4-induced liver fibrosis		
				*In vivo* (Liver fibrosis mouse model)			
Kang et al. (2022)	NASH	Human umbilical cord MSCs	Nrf2 ↑/NQO-1 ↑	*In vitro* (HucMSCs)	↓ Hepatic macrophage infiltration	100μg/mouse	Every 3 days from 14th to 20th week
				*In vitro* (HepG2 cells)	↑ Macrophage polarization		
				*In vitro* (AML-12 cells)	↓ Oxidative stress		
				*In vivo* (NASH mouse model)			
Zhang et al. (2022)	Ischemia–Reperfusion Intestine Injury	Bone marrow MSCs (miR-144-3p)	PTEN ↓/Akt ↑/Nrf2 ↑	*In vitro* (IEC-6 cells)	↓ Oxidative stress↓ Intestinal I/R Injury in a mouse model	50 µg	2 h
				*In vitro* (BMSCs)			
				*In vivo* (Ischemia-Reperfusion mouse model)*In vitro* (OGD/R) Cell Model)			
Cardiovascular System							
Xiao et al. (2022)	MI	Human bone marrow MSCs	Akt ↑/Nrf2 ↑/HO-1 ↑	*In vitro* (H9C2)		100 µL	Once every 3 days for a week
				*In vitro* (hBMSCs)	↓ Morphological damages in MI rat model		
				*In vivo* (MI rat model)	↓ Collagen deposition and infarction ratio		
				*In vitro* (Hypoxia cell model)			
Ning et al. (2021)	MI	Bone marrow MSCs (FNDC5)	Nrf2 ↑/HO-1 ↑	*In vitro* (BMSCs)	↓ Inflammatory responses	5–20 μg/mL	24 h
				*In vivo* (MI mouse model)	↓ Apoptosis		
					↑ Polarization of M2 macrophages		
					↑ Anti-inflammatory secretion		
Chen et al. (2022)	SAP-induced myocardial injury	hiPSC-MSCs	Nrf2 ↑/HO-1 ↑	*In vitro* (Hypoxia cell model)	↓ Oxidative stress	100 µg	Single dose
				*In vitro* (Cardiomyocytes)	↑ Cardiac function		
				*In vitro* (SAP rat model)	↑ Cell viability		
					↑ vWF and VEGF		
Xu et al. (2022b)	AF	Bone marrow MSCs	Nrf2 ↑/HO-1 ↑	*In vitro* (BMSCs)	↓ Heart Rhythm Changes	100 μg	48 h
				*In vitro* (AF rat model)	↓ Fibrosis Related Markers		
					↓ Apoptotic Cells and Inflammation		
Urinary System							
Cao et al. (2020)	Renal Ischemia-Reperfusion Injury	Human Placenta MSCs	Keap1 ↑/Nrf2 ↑	*In vitro* (hP-MSCs)	↑ Renal Function of AKI Mice	0.1 mL of 80 μg	Single dose (*in vivo*)
				*In vitro* (TECs)	↑ Preservation of Mitochondria in TECs		
				*In vivo* (AKI mouse model)			
Alzahrani (2019)	Renal Ischemia-Reperfusion Injury	Bone marrow MSCs (melatonin)	HO-1 ↑	*In vivo* (AKI mouse model)	↓ Oxidative stress	250 μg Exo	Single dose (*in vivo*)
				*In vitro* (BMSCs)			
Zhang et al. (2016)	Renal Ischemia-Reperfusion Injury	Human umbilical cord MSCs	Nrf2 ↑/ARE ↑	*In vitro* (hWJMSCs)	↓ Renal tubular injury	100 µg	24 h
				*In vitro* (Human foreskin fibroblasts)	↑ Renal function		
				*In vitro* (HucMSC)	↓ Cell apoptosis		
				*In vitro* (NRK-52)	↓ Oxidative stress		
				*In vivo* (AKI rat model)			
Skeletal System							
Yao et al. (2023)	GIOP	ADSCs	Nrf2 ↑/HO-1 ↑	*In vitro* (ADSCs)	↓ Apoptosis	50 and 100 μg	Single dose (*in vivo*)
				*In vitro* (MC3T3-E1)	↓ Oxidative damage		
				*In vivo* (GIOP rat model)	↓ Intracellular ROS generation		
					↓ Mitochondrial dysfunction		
					↑ Bone mass		
Zhang and Jin (2022)	OA	Bone marrow MSCs (lncRNA NEAT1)	miR-122-5p ↓/Sesn2 ↑/Nrf2 ↑	*In vitro* (HEK293T)	↑ Proliferation and Autophagy of Chondrocytes	10 μg	twice a week for 1 month
				*In vitro* (BMSCs)	↓ Apoptosis of Chondrocytes		
				*In vitro* (Human Chondrocytes)	↓ OA Progression		
				*In vivo* (OA Mouse Model)			
Yu et al. (2022)	IDD	Bone marrow MSCs (circ_0072464)	miR-431 ↓/Nrf2 ↑	*In vitro* (BMSCs)	↑ Alleviated IDD	200 μg	Every 2 days (*in vivo*)
				*In vitro* (Nucleus Pulposus Cells)	↑ Matrix Synthesis and Proliferation of Nucleus Pulposus Cells		
				*In vivo* (IDD Mouse Model)	↓ Nucleus Pulposus Cells Ferroptosis		
Shi et al. (2021)	IDD	Bone marrow MSCs	miR-155 ↑/Bach1 ↓/HO-1 ↑	*In vitro* (Rat Neural Progenitor Cells)	↑ Improving intervertebral disc degeneration	NM	NM
				*In vitro* (BMSCs)	↑ Autophagy in Neural Progenitor Cells		
				*In vitro* (OGD Model)	↓ Apoptosis		
Li et al. (2022b)	Tendon Injury	Bone marrow MSCs (Eugenol)	Nrf2 ↑/HO-1 ↑	*In vivo* (Tendon injury rat model)	↑ Tenogenesis	60 μg/mL	24h
				*In vitro* (BMSCs)	↑ Matrix regeneration		
				*In vitro* (TSCs)	↑ Fiber arrangement		
					↓ Oxidative stress		
Other Conditions							
Sun et al. (2023)	Diabetes	Human bone marrow MSCs	Nrf2 ↑/HO-1 ↑	*In vitro* (hBMSCs)	↓ HPMECs inflammation and ferroptosis	NM	24 h
				*In vitro* (HPMECs)			
Sun et al. (2022a)	Diabetic retinopathy	Human umbilical cord MSCs	NEDD4 ↑/PTEN ↓/AKT ↑/Nrf2 ↑	*In vitro* (HucMSC)	↓ Oxidative level	1 × 10^6^ particles suspended in 3 μL of PBS	Single dose (*in vivo*)
				*In vitro* (Human Fetal Lung Fibroblast 1)	↓ Retinal apoptosis		
				*In vivo* (Diabetic rat model)	↑ Proliferation ability of RPE cells		
				*In vitro* (Retinal pigment epithelium)			
Shan et al. (2022)	Peripheral Neuropathy	Bone marrow MSCs (SIRT1)		*In vitro* (BMSCs)	↑ Nerve conduction velocity	NM	NM
				*In vivo* (Diabetic rat model)	↓ Oxidative Stress		
					↓ DPN-Induced Mitochondrial Dysfunction		
Fu et al. (2023)	Biological root injury	hASCs	PI3K ↑/Akt ↑/Nrf2 ↑	*In vitro* (Dental Follicle Cells)	↑ Cell proliferation	160 μg/mL (*in vitro*)	24h
				*In vitro* (hASCs)	↑ Antioxidant capacity		
				*In vitro* (Dental Extracellular Matrix)	↑ Odontogenic and osteogenic differentiation		
				*In vivo* (Subcutaneous Implantation in Rats)	↓ Oxidative damage		
Gao et al. (2021)	PM2.5 exposure	Adipose MSCs	Nrf2 ↑	*In vitro* (Alveolar Type II Cells)	↓ Levels of ROS and inflammatory cytokines	10^9^ (*in vitro*)	NM
				*In vitro* (Alveolar Macrophages)	↑ M2-like macrophages		
				*In vitro* (PM2.5 exposed rat model)			

Not Mentioned (NM), Traumatic Brain Injury (TBI), Human umbilical cord mesenchymal stem cell (HucMSC), Spinal Cord Injury (SCI), Mesanchymal Stem Cell (MSC), Interleukin-1β (IL-1), Status epilepticus (SE), lipopolysaccharide (LPS), human neural stem cells (hNSC), Intracerebral hemorrhage (ICH), Delayed Neurocognitive Recovery (dNCR), human umbilical vein endothelial cells (HUVECs), endothelial progenitor cells (EPCs), cardiopulmonary bypass (CPB), reactive oxygen species (ROS), human amniotic mesenchymal stem cells (hAMSCs), Acute lung injury (ALI), acute respiratory distress syndrome (ARDS), Type II, alveolar epithelial cell (AECII), Cystic Fibrosis (CF), peripheral blood mononuclear cells (PBMCs), Oxygen-Glucose Deprivation/Reoxygenation (OGD/R), Human bone marrow mesenchymal stem cells (hBMSCs), Myocardial Infarction (MI), hiPSC-MSCs (Human induced Pluripotent Stem Cell-derived Mesenchymal Stem Cells), severe acute pancreatitis (SAP), von Willebrand Factor (vWF), vascular endothelial growth factor (VEGF), atrial fibrillation (AF), Human Placenta-Derived Mesenchymal Stem Cells (hP-MSCs), acute kidney injury (AKI), Tubular Epithelial Cells (TECs), hWJMSCs (human Wharton’s jelly-derived mesenchymal stem cells), adipose-derived stem cells (ADSCs), glucocorticoid-induced osteoporosis (GIOP), intervertebral disc degeneration (IDD), oxygen, Glucose, and Serum Deprivation (OGD), Tendon-Derived Stem Cells (TSCs), Human Pulmonary Microvascular Endothelial Cells (HPMECs), Diabetic peripheral neuropathy (DPN), Human Adipose-Derived Stem Cells (hASCs), Sulfur mustard (SM), ↑ (increased/activated), ↓ (decreased, inactivated).

### 3.1 Nervous system

#### 3.1.1 Traumatic injury

Traumatic Brain Injury (TBI) poses a substantial public health challenge in the United States, leading to elevated levels of illness and death. Effectively managing TBI requires a comprehensive, multidisciplinary approach that integrates medical, surgical, and psychological factors to improve patient outcomes (Capizzi et al., 2020). Long non-coding RNAs (lncRNAs) are a diverse class of RNA molecules that are longer than 200 nucleotides and do not code for proteins. They play crucial roles in gene regulation, chromatin remodeling, and cellular processes. Some lncRNAs function as molecular sponges for microRNAs (miRNAs), which are small non-coding RNAs that regulate gene expression by binding to target mRNAs. By binding to miRNAs, lncRNAs can sequester them away from their mRNA targets, preventing them from exerting their regulatory effects. This interaction between lncRNAs and miRNAs allows for fine-tuning of gene expression and contributes to the complexity of cellular regulatory networks (Chen et al., 2018; Chang et al., 2019; He et al., 2023). Human umbilical cord mesenchymal stem cell-derived exosomes (HucMSC-Exo) exhibit neuroprotective effects following TBI by engaging the lncRNA TUBB6/Nrf2 pathway. Administering HucMSC-Exo to a TBI mouse model reduces inflammation and ferroptosis induced by TBI, while simultaneously enhancing the expression of lncRNA TUBB6. This upregulation facilitates Nrf2 nuclear translocation, aiding in the mitigation of TBI-induced neuronal death. The importance of the TUBB6/Nrf2 pathway is emphasized by the partial reduction in neuroprotection observed upon TUBB6 knockdown. Furthermore, HucMSC-Exo suppresses ferroptosis-related markers such as ACSL4, and their modulation of the Nrf2 signaling pathway significantly contributes to alleviating oxidative damage and inflammation associated with TBI. These findings underscore the therapeutic potential of HUCMSC-Exo in managing TBI-related neurological complications (Zhang L. et al., 2024).

Spinal cord injury (SCI), another form of traumatic nervous system injury, presents a debilitating neurological condition characterized by acute and chronic phases. These phases are distinguished by destructive events such as ischemia, oxidative stress, inflammation, and apoptotic pathways. Various therapeutic strategies are aimed at alleviating neurodegeneration and reducing secondary neuronal damage. Ongoing efforts focus on developing neuroprotective and neuro-regenerative therapies (Anjum et al., 2020). EVs derived from melatonin-preconditioned MSCs enhance functional recovery in SCI by promoting M2-like microglia/macrophage polarization. Melatonin-preconditioned MSCs deliver ubiquitin-specific protease 29 (USP29) to regulate Nrf2 stability, inhibiting Nrf2 ubiquitination and degradation. This process upregulates HO-1 expression, influencing microglia/macrophage polarization and improving motor recovery. Additionally, melatonin reduces m^6^A RNA methylation of USP29, contributing to its upregulation (Liu W. et al., 2021; Li Y. et al., 2023).

In summary, the Nrf2/HO-1 pathway is pivotal in nervous system traumatic injuries by reducing oxidative damage and inflammation and promoting neuroprotection, contributing to potential therapeutic interventions in TBI and SCI.

#### 3.1.2 Inflammation injury

Inflammation, a crucial defense mechanism, responds to tissue injuries with peripheral cytokines (interleukin-1β, interleukin-6, tumor necrosis factor-α) influencing the brain and contributing to sickness responses (Wen and Zhang, 2023). This inflammatory response is implicated in neurodegenerative disorders in the central nervous system. Interleukin-1β (IL-1)-treated MSC-Exo significantly inhibited lipopolysaccharide (LPS)-induced astrogliosis and inflammatory responses in astrocytes. The IL-1-treated MSC-Exo reversed LPS-induced effects on calcium signaling, with the Nrf2 signaling pathway implicated in this reversal. It has been demonstrated that IL-1-treated MSC-Exo, through Nrf2 modulation, effectively suppressed inflammatory responses in astrocytes, both *in vitro* and in a status epilepticus mouse model. The findings suggest a potential therapeutic avenue for inflammatory brain conditions, emphasizing the role of the Nrf2 signaling pathway in mediating the anti-inflammatory effects of IL-1-treated MSC-Exo (Liu K. et al., 2021).

Additionally, inflammation is a pivotal player in nervous system injury. It triggers astrocyte activation and fosters their reactive states, leading to neurotoxicity and synaptic dysfunction observed across diverse neurological disorders. Xian et al. study investigated the therapeutic potential of MSC-Exo in addressing inflammation-induced astrocytic alterations and their effects on LPS-stimulated astrocytes and a status epilepticus mouse model. MSC-Exo attenuated reactive astrogliosis, inflammatory responses, aberrant calcium signaling, and mitochondrial dysfunction. The study revealed the involvement of the Nrf2-NFκB signaling pathway, indicating that MSC-Exo’s anti-inflammatory actions were mediated through Nrf2. Inhibition of Nrf2 weakened the protective effects, highlighting the pivotal role of Nrf2 and its downstream target, HO-1, in mitigating inflammation-induced astrocytic alterations (Xian et al., 2019).

These findings demonstrate that IL-1-treated MSC-Exo effectively mitigate inflammation-induced astrocytic alterations through the Nrf2 signaling pathway, providing potential therapeutic avenues for inflammatory conditions in the central nervous system.

#### 3.1.3 Hypoxia-reperfusion injury

Ischemia-reperfusion injury arises during stroke treatment involving reperfusion therapies, which seek to restore blood flow to the ischemic area. While vital for preventing neuronal damage, this process carries a significant risk due to subsequent tissue damage, termed ischemia-reperfusion injury. This injury stems from an imbalance in metabolic supply and demand, leading to mitochondrial dysfunction and neuronal death (Shen et al., 2021; Ge et al., 2023; Zhang X. et al., 2024).

When addressing neuronal hypoxia-reperfusion injury, extracellular vesicles derived from human neural stem cells (hNSC-EVs) showed promising protective effects. They reduced apoptosis, boosted neuronal survival, and promoted axonal elongation. Mechanistically, hNSC-EVs facilitated the translocation of Nrf2 to the nucleus, resulting in increased expression of antioxidant genes like HO-1. As a result, this process effectively decreased intracellular reactive oxygen species (ROS), shielding neurons from oxidative stress. These findings suggest that hNSC-EVs could be a valuable therapeutic approach for cerebral ischemia-reperfusion injury, potentially acting via the Nrf2/HO-1 pathway. Further *in vivo* investigations are necessary to confirm these results (Liu et al., 2020).

MSC-Exo demonstrated promising potential in an *in vitro* model mimicking cerebral ischemia-reperfusion injury (OGD/R; oxygen-glucose deprivation/reperfusion). It exhibited notable neuroprotective effects by attenuating OGD/R-induced neuronal apoptosis, reducing ROS production, and enhancing cell viability. A significant mechanism involved the modulation of Nrf2, with MSC-Exo effectively inhibiting OGD/R-induced Nrf2 accumulation. This regulatory effect coincided with increased activities of antioxidant proteins such as superoxide dismutase and glutathione peroxidase, highlighting the antioxidative potential of MSC-Exo. Moreover, MSC-Exo exerted a protective influence on mitochondrial function, preserving mitochondrial membrane potential and influencing the expression of key mitochondrial genes (DJ1, OPA1, Mfn-1, Mfn-2, LRRK2, and PINK). These collective findings suggest MSC-Exo as a promising therapeutic strategy for cerebral ischemia-reperfusion injury. Through its actions on oxidative stress, modulation of Nrf2-related antioxidant pathways, and preservation of mitochondrial function, MSC-Exo emerges as a potential regulator for future treatments in this pathological context (Guo et al., 2022).

In a separate study, researchers investigated the therapeutic potential of MSC-Exo enriched with miR-194 in a neurovascular endothelial cell injury model induced by OGD/R. The results revealed a decrease in miR-194 expression during OGD/R, and its upregulation mitigated OGD/R-induced injury in neurovascular endothelial cells (HBMECs). Mechanistically, miR-194 targeted and suppressed Bach1, a protein upregulated in OGD/R. Furthermore, Bach1 was found to bind to the 3′-UTR of Nrf2, suggesting a regulatory connection. Upregulating miR-194 elevated Nrf2 and HO-1 protein levels while inhibiting Bach1 in OGD/R-treated HBMECs. MSC-Exo enhanced cell viability and migration and provided protective effects against ferroptosis in OGD/R-injured HBMECs. Taken together, these findings suggest that miR-194-enriched MSC-Exos alleviate OGD/R-induced injury by targeting Bach1 and regulating the Nrf2/HO-1 signaling pathway, offering potential therapeutic benefits for cerebral injuries through the modulation of oxidative stress and ferroptosis (Li et al., 2021).

In summary, hNSC-EVs and miR-194-enriched MSC-Exo demonstrated promising therapeutic potential for cerebral ischemia-reperfusion injury by decreasing neuronal apoptosis, enhancing cell survival, and modulating the Nrf2/HO-1 signaling pathway. This suggests potential targets for future treatments in oxidative stress and ferroptosis-related cerebral injuries.

### 3.2 Other nervous system conditions

#### 3.2.1 Methotrexate-induced injury

Huang et al. investigated the effectiveness and underlying mechanisms of adipose-derived mesenchymal stem cell exosomes (ADSC-Exo) in mitigating methotrexate (MTX)-induced neuronal damage. They employed both *in vitro* models of H2O2-induced oxidative stress and an *in vivo* rat model of MTX-induced neuronal damage to evaluate the impact of ADSC-Exo on neuronal health and the Nrf2-ARE pathway. The results illustrated that ADSC-Exo successfully restored the structural and functional integrity of hippocampal neurons in MTX-treated rats. Activation of the Nrf2-ARE pathway alleviated oxidative stress, as evidenced by reduced ROS levels and enhanced cell viability. In the *in vitro* experiments, ADSC-Exo notably alleviated H2O2-induced oxidative stress in hippocampal neurons, indicating their antioxidative properties. The study further delineated the involvement of ADSC-exosomes in Nrf2-ARE pathway activation, revealing elevated protein expression of nuclear Nrf2, HO-1, and NAD(P)H quinone oxidoreductase 1 (NQO1) upon treatment with ADSC-Exo. Inhibition of Nrf2 using ML385, a specific Nrf2 inhibitor, mitigated the antioxidative effects of ADSC-Exo, underscoring the importance of the Nrf2-ARE pathway. In the *in vivo* MTX-induced neuronal damage model, ADSC-Exo exhibited a protective role by augmenting glutathione levels, reducing malondialdehyde content, and mitigating apoptosis of hippocampal neurons. Furthermore, ADSC-Exo attenuated the levels of pro-inflammatory cytokines (IL-6, IFN-γ, TNF-α) in brain tissue, suggesting an additional anti-inflammatory effect. (Huang et al., 2022). The findings collectively suggest that ADSC-Exo alleviates oxidative stress and neuronal damage induced by MTX by activating the Nrf2-ARE signaling pathway. This implies a potential therapeutic strategy for reducing the neurotoxic effects of MTX-induced neuronal damage.

#### 3.2.2 Vascular dementia

Vascular dementia (VaD), now referred to as vascular neurocognitive disorder, manifests as notable cognitive impairment directly associated with vascular brain injury. It encompasses various factors like atherosclerosis, arteriosclerosis, micro infarcts, and amyloid angiopathy, and its complex origin is frequently interconnected with neurodegenerative aspects. This underscores the significance of vascular elements and other factors in developing aging-related cognitive decline (Bir et al., 2021).

HucMSC-derived EVs (HucMSC-EVs) exhibited therapeutic potential in VaD by activating the PI3K/AKT/Nrf2 pathway. In a VaD rat model, HucMSC-EVs administration mitigated neurological impairment, improved cognitive function, and restored brain tissue structure. These protective effects were associated with reduced microglial M1 polarization, inflammation, and oxidative stress. HucMSC-EVs activated the PI3K/AKT/Nrf2 pathway in brain tissues, highlighting its crucial role in mediating the observed neuroprotection. When the PI3K pathway was inhibited, the beneficial effects of HucMSC-EVs on microglial polarization, inflammation, and oxidative stress were partially reversed, emphasizing the significance of the Nrf2 pathway in HucMSC-EVs-mediated neuroprotection in VaD (Wang et al., 2023).

#### 3.2.3 Amyotrophic lateral sclerosis

Amyotrophic lateral sclerosis (ALS), a fatal neurodegenerative disease affecting both upper and lower motor neurons, exhibits phenotypic heterogeneity, global CNS dysfunction, and genetic complexities. Limited therapeutic options challenge current management, but evolving diagnostic criteria, insights into pathophysiology, identification of biomarkers, and ongoing clinical trials offer prospects for improved care and outcomes, emphasizing ALS as a complex syndrome requiring a comprehensive interdisciplinary approach (Feldman et al., 2022).

MSC-EVs effectively reduced neurotoxicity in ALS astrocytes, diminishing their pathological activation and inflammatory characteristics. These EVs influence the nuclear translocation of Nrf2, promoting an enhanced antioxidant response in ALS astrocytes. This modulation is facilitated by specific microRNAs (miRNAs) carried by EVs, including miR-466q and miR-467f, which downregulate Mapk11 and facilitate Nrf2 nuclear translocation. The findings suggest that leveraging the Nrf2 pathway through EV-mediated regulation holds promise as a therapeutic strategy for ALS (Provenzano et al., 2022).

#### 3.2.4 Intracerebral hemorrhage

Intracerebral hemorrhage (ICH), a severe form of stroke predominantly resulting from small vessel diseases such as hypertensive arteriopathy or cerebral amyloid angiopathy, presents a substantial challenge due to the scarcity of effective treatments. Although recent progress in comprehending its causes, implementing acute interventions, and developing preventive approaches, including medical therapies and minimally invasive surgery, holds potential, the overall clinical prognosis remains unfavorable (Hostettler et al., 2019; Yang et al., 2023).

Exosomal miR-23b originating from bone marrow mesenchymal stem cells (BMSCs) sowed potential within a rat model of ICH. Administering exosomal miR-23b markedly diminished brain edema and enhanced behavioral outcomes in rats with ICH. Importantly, exosomal miR-23b effectively targeted microglia/macrophages in the region surrounding the hematoma. Additionally, the application of exosomal miR-23b exhibited potent antioxidant effects by reducing indicators of oxidative stress, including ROS and malondialdehyde, while boosting the activity of the antioxidant enzyme superoxide dismutase. The research identified the PTEN/Nrf2 pathway as a crucial mediator of antioxidant effects, showing increased nuclear translocation of Nrf2 and elevated levels of HO-1. These findings were confirmed through *in vitro* experiments using microglia BV2 cells treated with hemin to simulate conditions similar to ICH. Exosomal miR-23b was found to alleviate oxidative stress and inhibit NLRP3 inflammasome-mediated pyroptosis in these cell cultures, with the involvement of the PTEN/Nrf2 pathway once again implicated in these effects. In summary, exosomal miR-23b derived from BMSCs emerged as a promising therapeutic candidate for ICH. It demonstrated the ability to alleviate oxidative stress, hinder pyroptosis via NLRP3, and activate the PTEN/Nrf2 antioxidant pathway concurrently. These multifaceted neuroprotective properties suggest a potential avenue for the development of targeted therapies to improve outcomes in individuals with ICH (Hu et al., 2023).

#### 3.2.5 Neurocognitive recovery

Delayed neurocognitive recovery (dNCR) is a perioperative neurological complication marked by a significant deterioration in cognitive function, which impacts learning, memory, information processing, and attention. This phenomenon occurs 30 days after surgery, especially among older patients (Badenes et al., 2021).

The role of the MSC-Exo in addressing delayed dNCR in aged mice following exploratory laparotomy has been investigated. The findings indicated that prolonged escape latencies and altered spatial memory characterize surgery-induced cognitive impairment in aged mice. The examination of hippocampal tissues revealed evidence of ferroptosis in the dNCR group. Treating dNCR aged mice with MSC-Exo yielded positive results, ameliorating cognitive impairment. The protective effects were linked to the inhibition of hippocampal ferroptosis, as evidenced by improved mitochondrial morphology and modulation of key markers such as ROS, glutathione, malondialdehyde, Fe^2+^, GPX4, P53, and SLC7A11. Notably, the involvement of the Nrf2/HO-1 signaling pathway in mediating these effects was highlighted. SIRT1-dependent mechanisms played a crucial role in mediating the effects of MSC-Exo, with SIRT1 acting upstream to regulate Nrf2 and contribute to inhibiting hippocampal ferroptosis. In summary, MSCs-Exo demonstrated a therapeutic potential in alleviating cognitive impairment and inhibiting ferroptosis in aged mice experiencing dNCR. These effects were mechanistically linked to activating the Nrf2/HO-1 signaling pathway, highlighting the importance of antioxidant and neuroprotective mechanisms in addressing perioperative neurological complications (Liu et al., 2022).

#### 3.2.6 Seizure

In a study, Luo et al. raised evidence that MSC-EVs demonstrated potent antioxidant effects in countering oxidative stress-induced neuronal damage. The MSC-EVs, enriched with antioxidant miRNAs, exhibited remarkable restoration activities on hippocampal neurons subjected to oxidative insults *in vitro* and seizures *in vivo*. Notably, the Nrf2 signaling pathway was identified as a crucial mechanism underlying the therapeutic efficacy of MSC-EVs. The study found that MSC-EVs activated the Nrf2 defense system, leading to increased expression of HO-1. Additionally, the protective effects of MSC-EVs were compromised when Nrf2 was knocked down, highlighting the pivotal role of the Nrf2/HO-1 axis in mediating the antioxidative benefits of MSC-EVs in neurological disorders such as seizures (Luo et al., 2021).

#### 3.2.7 Summary

MSC-EVs overexpressing NRF2/HO-1 exhibit multifaceted neuroprotective properties across diverse types of nervous system injuries. Common mechanisms underlying their therapeutic effects include the modulation of inflammation, reduction of oxidative stress, promotion of neuronal survival, and enhancement of functional recovery. Specifically, MSC-EVs mediate their protective actions through the activation or stabilization of the Nrf2 signaling pathway, leading to the upregulation of antioxidant genes such as HO-1. This results in the mitigation of oxidative damage, suppression of neuroinflammation, and inhibition of apoptotic pathways, collectively contributing to neuronal preservation and functional improvement. These findings highlight the potential of MSC-EVs as promising therapeutic agents for various neurological conditions, offering a versatile approach to targeted intervention and neuroprotection.

### 3.3 Integumentary system

#### 3.3.1 Diabetic wounds

Diabetic wounds pose a significant and growing healthcare challenge globally. With approximately 500 million people affected by diabetes, diabetic wounds contribute to the staggering economic burden of over $300 billion in the United States alone. These wounds, characterized by persistent hyperglycemia and various complications, including infection, neuropathy, and impaired inflammatory responses, significantly impact patients' quality of life. Recurrent diabetic wounds often lead to costly treatments and amputations, emphasizing the urgent need for more effective diagnostic and therapeutic strategies (Malone-Povolny et al., 2019; Burgess et al., 2021; Li JM. et al., 2023). Regarding the role of HO-1 and Nrf2 in diabetic wound healing, several experiments have been conducted. MSC-Exo-derived HO-1 revealed exceptional ability to enhance the proliferation, migration, and angiogenic activities of fibroblasts, keratinocytes, and human umbilical vein endothelial cells (HUVECs), surpassing the effects of conventional exosomes. In an *in vivo* experiment using a diabetic mouse model, subcutaneous injection of MSC-Exo-derived HO-1 remarkably accelerated wound healing, fostering heightened tissue regeneration and vascularization (Cheng et al., 2024).

In another study, the role of circRNA-itchy E3 ubiquitin-protein ligase (circ-ITCH) in diabetic foot ulcer (DFU) models was investigated. Circ-ITCH downregulation was observed in DFU, and treatments involving exosomal circ-ITCH from BMSCs alleviated high glucose-induced ferroptosis and promoted angiogenesis in HUVECs by stabilizing Nrf2 mRNA and activating the Nrf2 signaling pathway. In the DFU mice model, exosomal circ-ITCH accelerated wound healing by inhibiting ferroptosis in a time-dependent manner. These findings highlight the potential therapeutic implications of targeting the circ-ITCH/Nrf2 axis in DFU (Chen et al., 2023).

Researchers isolated BMSCs and endothelial progenitor cells from rat bone marrow, harvested exosomes from BMSCs culture, and assessed their impact on endothelial progenitor cell tube formation. Additionally, diabetic rats with full-thickness wounds were treated with BMSC-Exo alone or in combination with Nrf2 knockdown or Nrf2 activation using tert-butylhydroquinone (TBHQ). The findings indicate that BMSC-Exo promoted endothelial progenitor cell tube formation, accelerated diabetic wound closure, re-epithelization, collagen deposition, and neovascularization while reducing local inflammation. Notably, the regenerative and anti-inflammatory effects of BMSC exosomes were influenced by Nrf2. Nrf2 knockdown hindered these effects, whereas tBHQ administration enhanced them. This suggests that Nrf2 plays a crucial role in mediating the therapeutic benefits of BMSC exosomes in diabetic wound healing (Wang et al., 2022). Compared to current conventional treatments for DFU therapy, such as wound debridement, negative pressure wound therapy, hyperbaric oxygen therapy (HBOT), wound dressing, and off-loading techniques, MSC-EV therapy offers several advantages and disadvantages. MSC-EVs provide a non-cellular approach to therapy, potentially reducing concerns related to immunogenicity and tumorigenicity associated with cell-based therapies. They are easier to store and administer, bypassing challenges such as cell viability and retention. Additionally, MSC-EVs possess paracrine signaling properties that contribute to their therapeutic effects, promoting tissue repair and angiogenesis. However, challenges remain in optimizing EV isolation, standardizing production methods, and determining optimal dosing regimens. Clinical trials investigating the clinical applicability of MSCs and their derivatives in DFU therapy have shown promising results. Autologous stem cell therapies, including bone marrow-derived MSCs (BM-MSCs), peripheral blood mononuclear cells (PBMNCs), adipose tissue-derived stromal vascular fraction (SVF), and allogeneic stem cell therapies such as human umbilical cord-derived MSCs (hUC-MSCs) and adipose tissue-derived MSCs (ADSCs), have demonstrated efficacy in promoting ulcer healing and reducing amputation rates. Studies have reported improved wound healing, angiogenesis, and transcutaneous oxygen tension in DFU patients treated with various types of MSCs. Additionally, clinical trials have shown that MSC-based therapies are safe, with minimal adverse effects reported. However, challenges remain, including the need for larger multi-center trials to validate efficacy, standardization of treatment protocols, and addressing potential side effects such as diarrhea, fever, and increased serum creatinine levels. Despite these challenges, MSC-based therapies offer a promising approach to DFU treatment and warrant further investigation to optimize their clinical utility. Therefore, while MSC-EV therapy shows promise as a novel approach in DFU therapy, further clinical studies are needed to establish its efficacy and safety before widespread clinical adoption (Reviewed in (Mahmoudvand et al., 2023; Yu et al., 2023)).

#### 3.3.2 Oxidative stress-induced skin injury

The main cause of skin injury is oxidative stress, which is triggered by ultraviolet irradiation and other damaging causes. This stress leads to the production of reactive oxygen species (ROS), which then damages proteins, lipids, and DNA in skin cells. Furthermore, it diminishes the functioning of antioxidant enzymes. The repercussions encompass sunburns, accelerated ageing, and an increased susceptibility to carcinogenesis. MSC-Exo exhibited protective benefits against skin damage caused by oxidative stress through the regulation of the Nrf2 defense system. These effects were indicated by the presence of positive markers (CD105, CD90, and CD73) and the ability to differentiate into different cell types. Both MSCs and MSC-Exo displayed typical characteristics of exosomes. Administration of MSC-Exo to keratinocytes stimulated with H2O2 leads to a notable decrease in the production of reactive oxygen species (ROS) and DNA harm, while simultaneously improving antioxidant functions. MSC-Exo alleviates aberrant calcium signaling and reinstates mitochondrial membrane potential in stressed keratinocytes. When MSC-Exo is administered in a mouse model of UV-induced skin injury, it reduces the thickness of the outer layer of the skin, enhances the density of collagen fibers, and decreases the levels of proinflammatory cytokines. Significantly, MSC-Exo reduces the expression of Nrf2 after oxidative stress, hence impacting the expression of genes involved in antioxidant activity. Reducing the activity of Nrf2 in a laboratory setting decreases the beneficial benefits of MSC-Exo, emphasizing the importance of Nrf2. Furthermore, the Nrf2 inhibitor ML385 partially diminishes the effects mediated by MSC-Exo *in vivo*. The results emphasize the important function of the Nrf2 defense system in facilitating the antioxidative and reparative characteristics of MSC-Exo. This indicates that MSC-Exo could be a promising therapeutic approach for skin disorders associated with oxidative stress (Wang et al., 2020).

### 3.4 Respiratory system

#### 3.4.1 Acute lung injury

Acute lung injury (ALI) is a disorder marked by the sudden onset of respiratory insufficiency. It is defined by symptoms including rapid breathing (tachypnea), acute lack of oxygen in the blood (hypoxemia), reduced lung flexibility (decreased lung compliance), and the presence of widespread infiltrates in the air sacs of the lungs (diffuse alveolar infiltrates). It frequently arises as a consequence of diverse lung traumas or systemic causes (Mokrá, 2020).

Zhang et al. examined the therapeutic efficacy of exosomes obtained from BMSC-Exo in treating ALI linked to cardiopulmonary bypass (CPB). Administration of BMSC-Exo significantly decreases histological lung damage, inflammatory cytokines, and oxidative stress in a rat model. BMSC-Exo significantly stimulates the Nrf2/HO-1 pathway while simultaneously suppressing NF-κB p65 and enhancing Akt/Nrf2/HO-1 signaling. BMSC-Exo has been found to inhibit the formation of reactive oxygen species (ROS) and inflammatory cytokines in alveolar macrophages through these routes, as demonstrated in in vitro investigations (Zhang et al., 2023).

In another study, the therapeutic potential of small EVs obtained from human amniotic mesenchymal stem cells (hAMSCs) engineered to overexpress Nrf2 was explored, aiming to protect against LPS-induced ALI in mice. After being exposed to LPS, the administration of Nrf2-sEVs resulted in a noteworthy decrease in lung damage, inflammation, and fibrosis, as indicated by the results. The protective benefits included reduced apoptosis, decreased infiltration of neutrophils and macrophages, and inhibition of the expression of pro-inflammatory cytokines. The Nrf2-sEVs were discovered to function by inhibiting the activation of the NLRP3 inflammasome and promoting the polarization of M2 macrophages. In addition, Nrf2-sEVs stimulated the Nrf2/HO-1 pathway, suggesting a possible antioxidant reaction. This study highlights the potential of Nrf2-sEVs as a therapeutic treatment for reducing lung injury caused by LPS. It emphasizes the role of Nrf2 in controlling inflammation and promoting tissue protection (Xu et al., 2022a).

Furthermore, Zhao et al. investigated the therapeutic efficacy of mesenchymal stem cell-derived extracellular vesicles (MSC-EVs) in the context of acute lung injury (ALI). The inhalation of MSC-EVs is more effective than injecting them into the tail vein. This method shows a decrease in pro-inflammatory cytokines, an increase in anti-inflammatory cytokines, and relief from ALI-induced pathology. In addition, MSC-EV therapy induces a shift in macrophage polarization towards an M2 phenotype, which is known for its anti-inflammatory properties. The Nrf2/HO-1 pathway activation is recognized as a vital mediator for the anti-inflammatory and antioxidant actions of MSC-EVs. The work highlights the importance of Nrf2 by showing that reducing Nrf2 levels decreases the anti-inflammatory and antioxidant effects. The results emphasize that inhaling MSC-EVs shows great potential as a treatment strategy for acute lung injury (ALI), with the Nrf2 protein playing a key role in regulating this process (Zhao R. et al., 2022).

Furthermore, the effectiveness of BMSC-Exo in reducing the severity of sepsis-induced acute respiratory distress syndrome (ARDS) via inhibiting cell death in type II alveolar epithelial cells (AECII) is shown. BMSC-Exo tackles the problem of mitochondrial dysfunction in AECIIs by increasing the expression of Nrf2, stimulating the production of new mitochondria, and preventing the fragmentation of existing mitochondria. The inhibitory effect of ML385 on Nrf2 hampers its protective action. In addition, BMSC-Exo significantly improves survival rates, decreases AECII apoptosis, reduces inflammatory cytokine levels, and alleviates lung damage in septic mice. These benefits are reversed by ML385. These findings reveal a new mechanism that demonstrates how BMSC-Exo, via activating Nrf2, has potential as a therapeutic intervention for lung damage caused by sepsis (Li Z. et al., 2023).

Furthermore, the therapeutic effectiveness of HucMSC-Exo in mitigating damage caused by excessive oxygen exposure in the lungs, heart, and kidneys of neonatal rats has been investigated. The delivery of HucMSC-Exo directly into the trachea has positive effects on the development of lung alveoli, the formation of new blood vessels, and the avoidance of enlargement of the right ventricle of the heart, anomalies in kidney function, and changes in the structure of the pulmonary blood vessels. The protective mechanisms involve a reduction in inflammatory cytokines, oxidative stress indicators, and changes in the transcriptome. HucMSC-Exo has a notable effect on increasing the expression of HO-1 and activating the JAK2/STAT3 signaling pathway. This suggests that HucMSC-Exo has the ability to improve the damage to several organs caused by excessive oxygen exposure (Dong et al., 2022).

Sulfur mustard, also known as 2,2‐dichlorodiethyl sulfide or SM, is a highly strong chemical that can cause significant damage to various organs and systems in the body, such as the respiratory system, skin, and eyes. It is a constant threat in warfare and terrorism. HucMSC-Exo exhibited therapeutic characteristics in lung injury induced by SM. The experiments conducted on mice exposed to SM demonstrate that HucMSC-Exo, namely, its miR-199a-5p component, greatly improves survival rates, mitigates lung damage, and decreases oxidative stress and apoptosis. Functionally, miR-199a-5p enhances the activation of the Nrf2 signaling pathway, resulting in the increased expression of antioxidant enzymes HO1 and NQO1. The study proposes that HucMSC-Exo, through the action of miR-199a-5p, reduces the harmful effects of oxidative stress caused by SM by regulating the CAV1/NRF2/HO1 pathway. This highlights prospective treatment options for lung injuries caused by SM. CAV1 acts as a suppressor of antioxidant enzymes by directly suppressing the production of Nrf2 (Gong et al., 2023).

The route of administration of MSC-EVs significantly influences their therapeutic efficacy in treating ALI. When administered intravenously, MSC-EVs are distributed systemically, potentially allowing for a broad targeting of injured tissues, but they may also be rapidly cleared by the mononuclear phagocyte system, predominantly in the liver and spleen, which could reduce their availability and efficacy in the lung tissue. In contrast, intratracheal instillation delivers MSC-EVs directly into the lung, enhancing their local concentration and immediate interaction with the pulmonary tissues. This localized delivery can lead to more efficient utilization of the vesicles, potentially reducing inflammation and promoting repair more effectively than systemic administration. MSC-EVs administered via inhalation not only efficiently target lung tissues but also offer superior anti-inflammatory effects and enhanced tissue repair in ALI mice compared to tail vein injection. The findings suggest that inhalation could be a more effective delivery route for MSC-EVs in treating pulmonary conditions such as ALI, potentially due to the direct delivery and higher concentrations reaching the affected site. These results could pave the way for developing targeted therapies for respiratory ailments using MSC-EVs (Zhao R. et al., 2022). Thus, the choice between systemic versus local delivery can be crucial, depending on the specific therapeutic goals and the nature of the lung injury. Although promising results have been observed across all methods in this review, it is highly recommended that future research compare these routes to clarify the most effective method of administration (Tolomeo et al., 2023).

To summarize, these studies consistently emphasize the significant therapeutic potential of MSC-EVs and sEVs in treating acute lung injury (ALI) caused by different factors. These vesicles demonstrate significant anti-inflammatory, antioxidant, and tissue-protective properties. The stimulation of the Nrf2/HO-1 pathway is a key mechanism that plays a vital role in mediating these therapeutic effects. Regardless of whether they are produced from bone marrow or amniotic mesenchymal stem cells, these vesicles have shown effectiveness in many models, such as CPB-associated acute lung injury, LPS-induced lung injury, and sepsis-related acute respiratory distress syndrome. Furthermore, the results emphasize the significance of different methods of administration, with inhalation being particularly effective.

#### 3.4.2 Cystic fibrosis

Cystic fibrosis (CF) is a hereditary condition that affects multiple organs, particularly the respiratory and digestive systems. It is characterized by anomalies in chloride channels. Current therapies include on the use of pharmaceuticals, physiotherapy, and nutritional support. Ongoing research is exploring the use of protein rectifiers, gene therapy, and new therapeutics to alleviate symptoms. Although there has been improvement, there are still obstacles that remain, such as the lack of effective treatments for younger patients, complications connected to genetic mutations, the role of genetics on the condition, possible interactions between drugs, negative side effects, and economic considerations. Continued investigation of groundbreaking approaches remains essential (Radlović, 2012; Rafeeq and Murad, 2017; He et al., 2020).

Human lung MSC-EVs exhibits anti-inflammatory effects by decreasing the concentrations of pro-inflammatory cytokines (IL-1β, IL-8, and IL-6) in cystic fibrosis airway epithelial cells during inflammation produced by TNFα. In addition, EV therapy improves the antioxidant defenses of CF cells, as seen by increased Fe2+ levels. The work reveals the role of the peroxisome proliferator-activated receptor gamma (PPARγ), a transcription factor that has antioxidant and anti-inflammatory properties, and its downstream regulators, the NF-kB/HO-1 axis, in the mechanism. The results indicate that EVs obtained from lung MSCs show potential in reducing excessive inflammation in CF, with the involvement of HO-1 in coordinating these effects (Zulueta et al., 2018).

### 3.5 Digestive systm

#### 3.5.1 Liver ischemia-reperfusion injury

Liver ischemia-reperfusion injury is a major issue in surgical procedures such as hepatic resection and liver transplantation. It causes graft dysfunction and post-transplantation liver failure. The challenge of effectively managing this condition is due to inflammation and complex molecular processes (Peralta et al., 2013; Jiménez-Castro et al., 2019). Zuo et al. address the issue of donor shortages in liver transplantation by investigating the protective effects of HO-1-modified BMSCs and their small EVs in the context of ischemia-reperfusion injury in transplanted steatotic liver mouse models. The findings revealed that both BMSCs and BMSC-EVs can inhibit ferroptosis, a mechanism associated with cell death in liver ischemia-reperfusion injury. The study highlights miR-214-3p in BMSC-EVs as a crucial factor targeting cyclooxygenase 2 (COX2) to suppress ferroptosis. This innovative approach suggests a promising strategy for alleviating liver ischemia-reperfusion injury in steatotic transplanted livers and addresses the challenge of donor liver shortages (Zuo et al., 2023). Also, Li et al. conducted a similar study investigating the protective effects of HO-1-modified BMSCs in steatotic liver ischemia-reperfusion injury models by targeting ferroptosis. The results demonstrate that HO-1-modified BMSCs alleviate liver damage and inhibit ferroptosis by modulating the IREB2/FTH1/TFR1 pathway. HO-1-modified BMSCs, rich in miR-29a-3p, are crucial in reducing intracellular Fe^2+^, suppressing ferroptosis, and protecting against steatotic liver ischemia-reperfusion injury. The findings provide valuable insights into the therapeutic potential of HO-1-modified BMSCs and their exosomes, highlighting the role of HO-1 in mitigating oxidative stress and ferroptosis in the context of liver ischemia-reperfusion injury (Li et al., 2022a).

Moreover, HO-1-modified BMSC-Exo alleviates liver ischemia-reperfusion injury by delivering miR-124-3p, downregulating the ferroptosis regulator, prostate six transmembrane epithelial antigen 3 (STEAP3). This reduces lipid peroxidation and mitigates liver damage in steatotic grafts. The findings highlight the potential therapeutic role of HO-1-modified BMSC-Exo in liver transplantation, offering insights into addressing donor liver shortages (Wu et al., 2022).

Additionally, Sun et al. investigated the protective effects of combining xenogenic ADSC-Exo with melatonin against liver ischemia-reperfusion injury. The combined treatment demonstrated superior outcomes over ADSC-Exo alone, significantly reducing inflammation, apoptosis, and oxidative stress compared to individual treatments. Specifically, the expression of HO-1 showed a progressive increase from the control to the combined treatment group. This suggests a key role for HO-1 in the observed additive protective effect, emphasizing its potential as a therapeutic target for acute liver ischemia-reperfusion injury (Sun et al., 2017).

These studies highlight the innovative approaches using HO-1-modified MSCs and their EVs to address liver ischemia-reperfusion injury. These interventions demonstrated protective effects against ferroptosis, particularly in steatotic livers, addressing challenges related to donor shortages in transplantation. Insights into molecular mechanisms and pathway modulation, including IREB2/FTH1/TFR1 and microRNA-124-3p delivery, provide valuable understanding. The findings suggest promising strategies to mitigate oxidative stress, inflammation, and ferroptosis, emphasizing the therapeutic potential of targeting the Nrf2 and HO-1 pathways in acute liver injury. Combinatorial treatments, like xenogenic adipose mesenchymal stem cell-derived exosomes with melatonin, show additive protective effects. Overall, these studies contribute valuable knowledge for developing effective clinical strategies for liver ischemia-reperfusion injury.

#### 3.5.2 Acute liver injury

Acute liver failure is a severe condition marked by sudden hepatocyte injury, manifesting with a rapid increase in aminotransferases, changes in mental function, and disrupted coagulation. It is typically induced by diverse factors like paracetamol toxicity, hepatic ischemia, viral and autoimmune hepatitis, as well as drug-induced liver injury (Stravitz and Lee, 2019).

Baicalin-pretreated MSC-Exo (Ba-Exo) demonstrated a significant protective effect against acute liver injury by activating the Keap1/Nrf2 pathway, Ba-Exo attenuated liver damage, reduced inflammation, and inhibited lipid peroxide-induced ferroptosis in hepatocytes. The elevated P62 levels in Ba-Exo activates the Keap1/NRF2 pathway. Inhibition of the Nrf2 pathway reverses the ferroptosis-inhibiting effects of Ba-Exo (Zhao S. et al., 2022). Comparing the therapeutic efficacy of MSC-EVs from various sources, including umbilical cord, adipose tissue, and bone marrow, is crucial for optimizing the selection of cell sources in regenerative medicine. Research indicates that MSC-EVs exhibit regenerative potential similar to their parent cells, making them promising candidates for cell-free therapies. Studies have demonstrated differences in the cargo and biological properties of MSC-EVs derived from different tissue sources, suggesting that their therapeutic efficacy may vary. For instance, MSC-EVs from umbilical cord tissue have been shown to possess superior immunomodulatory and regenerative properties compared to those from adipose tissue and bone marrow. Additionally, the abundance of MSC-EVs in umbilical cord tissue, coupled with non-invasive isolation methods, makes them an attractive and accessible cell source for therapeutic applications (Jin et al., 2013).

#### 3.5.3 Liver fibrosis

Liver fibrosis is a pathological state characterized by the long-term accumulation of extracellular matrix components, mainly caused by hepatic myofibroblasts. This mechanism is a common feature in the progression of chronic liver disorders, regardless of their underlying etiology. Fibrosis development entails inflammatory responses and wound healing mechanisms, which affect illness prognosis and increase the risk of hepatocellular carcinoma. Although progress has been made in understanding the possibility of reversing liver fibrosis by targeting the underlying cause, finding effective treatments for fibrosis in clinical practice is still a significant and unresolved challenge (Kisseleva and Brenner, 2021; Pan et al., 2022).

Liu et al. explored the therapeutic potential of EVs derived from human-induced hepatocytes (hiHep-EVs) in liver fibrosis. These EVs effectively diminish inflammatory gene expression, inhibit hepatic stellate cell activation via the TGF-β1/Smad signaling pathway, and mitigate oxidative stress, inflammation, and fibrosis in a CCl4-induced liver fibrosis mouse model. The experiments showed that the hiHep-EVs and MSC-EVs have similar effects. Significantly, hiHep-EVs activate the Nrf2/HO-1 signaling pathway, augmenting antioxidant responses and mitigating liver fibrosis. The study suggests that hiHep-EVs present a promising therapeutic role for liver fibrosis, underscoring their ability to modulate Nrf2 and HO-1 for potent anti-fibrotic effects (Liu W. et al., 2023). In addition, adipose tissue-derived mesenchymal stem cells (AT-MSCs) and umbilical cord-derived mesenchymal stem cells (UC-MSCs) exhibit distinct *ex-vivo* expansion characteristics, with AT-MSCs displaying faster attachment, proliferation, and higher population doublings compared to UC-MSCs. However, UC-MSCs demonstrate superior self-renewal capacity, maintaining proliferation rates even at later passages. Phenotypically, both MSC types express characteristic MSC markers and pluripotent genes, with slight differences in expression levels. While both AT-MSCs and UC-MSCs demonstrate multilineage differentiation potential, AT-MSCs exhibit a higher capacity for adipogenic and osteogenic differentiation. In therapeutic efficacy against hepatic fibrosis, both MSC types effectively reduce liver fibrosis in preclinical models, with similar improvements in histopathological features and suppression of fibrosis-related gene expression. These findings underscore the potential of both AT-MSCs and UC-MSCs as promising treatments for hepatic fibrosis, albeit with nuanced differences in their expansion characteristics and differentiation potentials (Ghufran et al., 2024).

#### 3.5.4 Non-alcoholic steatohepatitis

Non-alcoholic steatohepatitis (NASH) is an advanced stage of non-alcoholic fatty liver disease (NAFLD), marked by hepatic steatosis, lipid accumulation, and subsequent inflammation and hepatocyte injury. It can progress to fibrosis, cirrhosis, and hepatocellular carcinoma. Factors like lipotoxicity, oxidative stress, and immune dysregulation contribute, with dysregulated metabolic pathways and immune cell-mediated processes playing key roles. Treatment involves lifestyle changes, weight reduction, and pharmacological options like vitamin E and pioglitazone. Ongoing research explores novel therapies to address NAFLD’s diverse clinical manifestations (Paternostro and Trauner, 2022).

Both *in vivo* and *in vitro* investigations revealed that HucMSC-Exo mitigated NASH by reducing liver fat accumulation, inflammation, and oxidative stress. The observed protective effects were linked to the elevation of the Nrf2/NQO-1 antioxidant signaling pathway. Exosome treatment boosted Nrf2 activation, resulting in increased expression of NQO-1 and other antioxidant enzymes. Additionally, the involvement of AMPK in this mechanism has been highlighted. These results underscore the potential therapeutic significance of HucMSC-Exo exosomes, underscoring the Nrf2/NQO-1 pathway’s role in alleviating oxidative stress and inflammation in NASH (Kang et al., 2022).

#### 3.5.5 Intestine ischemia-reperfusion injury

Intestinal ischemia/reperfusion injury is a critical and potentially fatal condition that causes damage to the small intestine as a result of the obstruction and subsequent restoration of blood flow. This lesion impacts both the tissue of the intestines and the circulatory system, specifically affecting the small blood vessels and the vessels in the mesentery. The harmful consequences predominantly manifest in the apical villi of microvessels, leading to reduced blood flow in the intestinal mucosa. These effects are connected to alterations in gene expression that are associated with inflammation, apoptosis, and cell proliferation. Accurate clinical evaluation and appropriate treatments require a thorough comprehension of the dynamic nature of this condition (Gordeeva et al., 2017).

The BMSC-Exo treatment shows promise in mitigating the harm caused by decreased blood flow and subsequent recovery in the intestines. This is achieved by regulating the imbalance of reactive oxygen species through the miR-144-3p/PTEN/Akt/Nrf2 pathway. BMSC-Exo alleviates histopathological damage, reduces apoptosis, and enhances antioxidant defenses in both *in vivo* and *in vitro* models of intestinal ischemia/reperfusion injury. Importantly, miR-144-3p in BMSC-Exo selectively focuses on PTEN, leading to the decrease in its expression. The PTEN/Akt/Nrf2 signaling pathway is activated, leading to increased synthesis of Nrf2 and its consequent antioxidant enzyme HO-1 (Zhang et al., 2022).

### 3.6 Cardiovascular system

#### 3.6.1 Myocardial infarction

Myocardial infarction (MI) is the result of arterial plaques that constrict blood flow to the heart, leading to damage of the heart muscles. Indications include discomfort in the chest, difficulty breathing, and feelings of sickness, necessitating rapid medical interventions. Prevention involves making changes to one’s lifestyle. While advancements in understanding and treating heart attacks have improved survival rates, there are still persistent difficulties in identifying and preventing heart attacks, which remain a leading cause of cardiovascular deaths globally (Lu et al., 2015).

The research findings showed that BMSC-EVs increased the survival rate of cardiomyocytes, increased the expression of vWF and VEGF, and activated the Akt/Nrf2/HO-1 pathway, resulting in overall improvements in outcomes for myocardial infarction (MI). Significantly, the overexpression of zinc finger antisense 1 (ZFAS1) was found to be a critical determinant, as it effectively counteracted the beneficial effects of extracellular vesicles (EVs). The results indicate that EVs have the potential to reduce damage caused by myocardial infarction (MI) by inhibiting the expression of ZFAS1 and promoting the Akt/Nrf2/HO-1 pathway. This provides a new approach for the treatment of MI (Xiao et al., 2022).

Fibronectin type III domain-containing protein 5 (FNDC5)-preconditioned MSC-Exo (FNDC5-MSC-Exo) demonstrated improved anti-inflammatory characteristics and promoted M2 macrophage polarization in comparison to MSC-Exo alone in experimental models. FNDC5-MSC-Exo exhibited a decrease in pro-inflammatory cytokines, an increase in anti-inflammatory cytokines, and promoted the polarization of M2 macrophages in laboratory settings. The underlying process involved the blocking of the NF-κB signaling route and the increase of the Nrf2/HO-1 Axis. This study indicates that FNDC5-MSC-Exo has potential as a cell-free therapy strategy for MI, with Nrf2 and HO-1 playing crucial roles in its anti-inflammatory actions and regulation of macrophages (Ning et al., 2021).

In addition, researchers discovered that exosomes obtained from mesenchymal stem cells produced from human-induced pluripotent stem cells (HiMSC-Exo) improved the survival of heart muscle cells and alleviated myocardial infarction caused by severe acute pancreatitis (SAP) through the activation of the Akt/Nrf2/HO-1 signaling pathway. This activation enhanced heart function, decreased infarction, and inhibited oxidative stress. The findings emphasizes the crucial importance of the Nrf2/HO-1 axis in facilitating the positive effects of Exo. This suggests that manipulating this route could be a feasible therapeutic approach for treating myocardial infarction generated by severe acute pancreatitis (Xiao et al., 2022).

#### 3.6.2 Atrial fibrillation

Atrial fibrillation (AF), the most common irregular heartbeat worldwide, is linked to a higher likelihood of mortality, stroke, and peripheral embolism. It affects approximately 33.5 million people and can be caused by factors such as changes in the electrical and structural properties of the atrial tissue, a sedentary lifestyle, dietary habits, and potential genetic factors (Sagris et al., 2021).

Xu et al. conducted an *in vivo* investigation to examine the involvement of Nrf2 and HO-1 in atrial fibrillation (AF) by utilizing exosomes generated from BMSCs from rats. The expression of Nrf2 and HO-1 was dramatically reduced in the myocardium of rats with AF. Administering exosomes produced from BMSCs that overexpress Nrf2 reduced the duration of AF, decreased cardiomyocyte apoptosis, and suppressed atrial fibrosis and inflammation in rats. The results indicate that activating the Nrf2/HO-1 pathway using exosomes produced from bone marrow mesenchymal stem cells (BMSCs) could be a promising treatment approach for AF. This approach could help reduce abnormal heart rhythms, myocardial fibrosis, cell death, and inflammation (Xu et al., 2022b).

### 3.7 Urinary system

#### 3.7.1 Renal ischemia-reperfusion injury

Renal ischemia/reperfusion injury, commonly observed in situations like kidney transplantation or cardiovascular surgery, occurs due to insufficient oxygen supply to the renal tissue, leading to increased death rates. The complex mechanisms responsible for this phenomenon are examined using many experimental models, ranging from cell lines cultured in a laboratory to surgical procedures conducted in living organisms. The intricacy of the situation highlights the necessity for additional investigation to improve understanding and investigate new treatment options. (Shiva et al., 2020).

Cao et al. used a new aggregation-induced emission luminogen, DPA-SCP, to noninvasively monitor MSC-EVs in renal ischemia-reperfusion injury. MSC-EVs accumulated in damaged kidney tubules and exhibited regenerative effects by activating the Keap1/Nrf2 axis. Specifically, miRNA-200a-3p, transported by MSC-EVs, triggered the Keap1-Nrf2 pathway, resulting in heightened expression of Nrf2 and the antioxidant protein SOD2. This activation played a pivotal role in maintaining the structure and function of mitochondria, ultimately contributing to the recovery of the kidney. The study underscores the importance of Nrf2 and HO-1 in the protective mechanisms of MSC-EVs against oxidative stress and inflammation in cases of acute kidney injury (Cao et al., 2020).

Alzahrani conducted a study to investigate the therapeutic effects of melatonin-preconditioned MSC-Exo on renal ischemia-reperfusion injury. The melatonin-preconditioned MSC-Exo treatment shown greater protective benefits in comparison to both MSCs and non-preconditioned MSC-Exo. The observed benefits were decreased kidney injury, improved renal function, decreased oxidative stress, suppressed cell death, reduced inflammation, accelerated tissue regeneration, and increased blood vessel formation. The treatment’s efficacy was notably associated, to some extent, with the activation of the antioxidant pathway HO-1. This study proposes that the use of melatonin-preconditioned MSC-Exo treatment has the potential to reduce renal ischemia-reperfusion injury by utilizing HO-1-mediated antioxidant responses (Alzahrani, 2019).

Furthermore, EVs obtained from mesenchymal stromal cells present in the gelatinous substance surrounding the vessels of the umbilical cord, known as Wharton’s Jelly, demonstrated a defensive impact against acute kidney injury caused by ischemia-reperfusion injury. The MSC-EVs exhibited the capacity to mitigate damage to the renal tubules, improve renal function, and reduce cell apoptosis and injury markers. Significantly, these EVs were discovered to boost the activation of Nrf2 and increase the expression of HO-1, suggesting a vital function in stimulating anti-oxidative responses. This study suggests that MSC-EVs can effectively alleviate acute kidney injury by influencing the Nrf2/ARE pathway. This provides valuable information about the mechanism behind MSC-EV therapy for AKI (Zhang et al., 2016).

In summary, these investigations underline possible therapeutic strategies for renal ischemia-reperfusion injury. Using MSC-EVs revealed regeneration effects through the Keap1-Nrf2 pathway and activation of antioxidant mechanisms. Melatonin-preconditioned MSC-Exo treatment revealed improved protection against renal ischemia-reperfusion injury, decreasing kidney injury and engaging antioxidant mechanisms. Also, MSC-Exo displayed a protective impact on ischemia-reperfusion damage-induced acute kidney injury by relieving renal tubular injury and activating the Nrf2/ARE pathway. These findings provide vital insights into prospective therapeutic techniques and the underlying processes to reduce renal ischemia-reperfusion injury, offering hope for improved outcomes in cases of acute kidney injury.

### 3.8 Skeletal system

#### 3.8.1 Osteoporosis

Glucocorticoid-induced osteoporosis (GIOP) is a frequently occurring complication caused by long-term and high-dose use of glucocorticoids. It is characterized by increased bone breakdown, reduced bone formation, and a higher likelihood of fractures due to an initial but temporary imbalance between bone formation and breakdown. Holistic management include the assessment of fracture risk, the implementation of non-pharmacological interventions for all patients, and the consideration of pharmacological options (Lane, 2019).

*In vitro* analysis showed that dexamethasone-induced oxidative stress and apoptosis in osteoblasts were alleviated by ADSC-Exo, which restored osteogenic potential and reduced ROS generation. Notably, ADSC-Exo upregulated Nrf2 and its downstream effector HO-1, which is critical in combating oxidative damage. In a GIOP rat model, ADSC-Exo exhibited therapeutic effects by inhibiting apoptosis, preserving bone mass, and enhancing Nrf2, HO-1, and osteogenic marker expressions. This suggests that ADSC-Exo holds promise in countering GIOP through Nrf2/HO-1-mediated antioxidative and osteoprotective pathways. However, there may be potential differences in the mechanisms of action between these two environments. *In vitro* studies have shown that MSC-EVs alleviate oxidative damage, promote osteogenic differentiation, and inhibit apoptosis in osteoblasts, primarily through the activation of pathways such as Nrf2/HO-1. In contrast, *in vivo* experiments have demonstrated additional complexities, including interactions with the immune system, biodistribution, and targeting of specific cell populations within the bone microenvironment. To optimize the preparation and administration of MSC-EVs for improved *in vivo* efficacy, several factors need consideration. These include standardizing isolation and characterization techniques to ensure consistent EV quality, optimizing delivery routes to enhance EV targeting and retention in bone tissues, and exploring strategies to modulate EV cargo for enhanced therapeutic potency. Moreover, understanding the dynamic interplay between MSC-EVs and the complex *in vivo* environment, including the immune response and tissue remodeling processes, is crucial for developing effective MSC-EV-based therapies for osteoporosis (Yao et al., 2023).

#### 3.8.2 Osteoporosis

Osteoarthritis, the most common joint disorder primarily impacting diarthrodial joints, exhibits a complex pathophysiology influenced by factors such as age, obesity, and multifactorial elements, resulting in mutual destruction. Existing treatments generally focus on alleviating symptoms, and despite ongoing progress, the lack of an authorized drug that can modify the condition highlights the need for improved methods of detecting and assessing risk, as well as more tailored therapeutic approaches (Martel-Pelletier et al., 2016).

The study conducted by Zhang et al. focused on examining the therapeutic potential of BMSC-EV harboring lncRNA NEAT1 in osteoarthritis (OA). BMSC-EVs supplied NEAT1, which activated the Sesn2/Nrf2 axis through binding to miR-122-5p. This activation promoted chondrocyte proliferation and autophagy, while simultaneously suppressing apoptosis. NEAT1-BMSC-EVs demonstrated a mitigating effect on the progression of OA in a mouse model. This was observed through a decrease in cartilage degradation and apoptosis. The NEAT1/miR-122-5p/Sesn2/Nrf2 axis emerges as a crucial regulatory pathway, suggesting BMSC-EV-mediated NEAT1 delivery as a promising strategy for OA treatment by modulating NRF2 and HO-1 signaling pathways. *In vitro* studies demonstrate the ability of MSC-EVs to effectively deliver therapeutic cargo, such as NEAT1, to chondrocytes, promoting proliferation, autophagy, and suppressing apoptosis. However, transitioning to an *in vivo* environment presents distinct challenges and complexities. *In vivo*, MSC-EVs encounter a dynamic milieu with various biological barriers, including immune responses, clearance mechanisms, and tissue-specific microenvironments, which may influence their efficacy. Additionally, factors such as EV biodistribution, stability, and targeting specificity become crucial considerations. To optimize the preparation and administration of MSC-EVs for enhanced *in vivo* efficacy in treating OA, strategies such as engineering EVs with surface modifications for targeted delivery to affected joints, optimizing EV cargo loading to enhance therapeutic potency, and exploring novel delivery routes (e.g., intra-articular injection, systemic administration) can be employed. Furthermore, advancements in EV isolation, purification, and characterization techniques are essential to ensure the reproducibility and scalability of MSC-EV-based therapies. Integrating these approaches will facilitate the translation of MSC-EV therapy from bench to bedside, offering promising prospects for improving OA management in clinical settings (Zhang and Jin, 2022).

#### 3.8.3 Disc degeneration

Intervertebral disc degeneration is a complex condition characterized by an imbalance between anabolic and catabolic processes, resulting in changes to the extracellular matrix, loss of nucleus pulposus cells, oxidative stress, and inflammation. This condition is influenced by factors such as aging, mechanical trauma, lifestyle choices, and genetic abnormalities, and it has significant economic consequences. Although current treatments are limited, there is potential in using stem cell-derived exosomes and regenerative therapies to address the degenerative process (Krut et al., 2021).

The researchers examined the function of circ_0072464, which was transported by BMSC-EV, in reducing intervertebral disc degeneration (IDD). The researchers discovered that the presence of BMSC-EV-loaded circ_0072464 hindered the process of ferroptosis in nucleus pulposus cells (NPC) by increasing the levels of Nrf2 through a competitive interaction with miR-431. This approach facilitated the production of matrix and the proliferation of neural progenitor cells (NPCs), ultimately alleviating intervertebral disc degeneration (IDD) in both laboratory experiments and living organisms. The results indicate that the circ_0072464/miR-431/Nrf2 pathway could be a promising target for treating IDD, emphasizing the importance of Nrf2 in reducing ferroptosis and enhancing disc health (Yu et al., 2022).

Furthermore, the work conducted by Shi et al. revealed that the combination of BMSCs and NPCs in a co-culture setting resulted in noteworthy therapeutic benefits for IDD. The protective strategy entails the enhancement of miR-155 expression in NPCs by the use of exosomes generated from BMSCs. Increased levels of miR-155 specifically target Bach1, resulting in the upregulation of HO-1 expression via the Nrf2 pathway. This leads to an increase in autophagy and a decrease in apoptosis in NPCs. As a result, this procedure reduces disc degradation by enhancing the survival of cells and preventing degenerative alterations (Shi et al., 2021).

#### 3.8.4 Tendon injury

Li et al. explored the protective function of eugenol-preconditioned extracellular vesicles obtained from bone marrow mesenchymal stem cells (EUG-BMSC-EVs) against damage caused by oxidative stress in tendon stem cells (TSCs). EUG-BMSC-EVs successfully mitigated the detrimental effects of H2O2 on TSC viability, tenogenic differentiation, and apoptosis levels. The activation of the Nrf2/HO-1 pathway by EUG-BMSC-EVs increased the antioxidant capacity and reduced oxidative stress in TSCs. In a model of rat patellar tendon injury, TSCs that were pretreated with EUG-BMSC-EV showed greater ability to generate tendon tissue, regenerate the extracellular matrix, and arrange the fibers. This highlights the potential of activating the Nrf2/HO-1 pathway to enhance the healing of tendons. The findings suggest that EUG-BMSC-EVs could be a promising therapeutic approach for tendon healing by reducing oxidative stress through the regulation of Nrf2/HO-1 (Li et al., 2022b).

### 3.9 Other systems

#### 3.9.1 Diabetes-related conditions

Epoxyeicosatrienoic acids (EETs) play a critical role in controlling MSCs and have promising therapeutic implications for diabetes. EETs, which are produced by the CYP450 monooxygenase system, play a role in reducing body fat and improving insulin sensitivity in MSCs, partially by increasing the expression of heme oxygenase-1 (HO-1). The positive feedback loop entails the activation of AMPK and pAKT pathways by EET, which leads to the suppression of soluble epoxide hydrolase and subsequent overexpression of HO-1. EETs decrease the expression of adipogenic markers and simultaneously enhance the levels of HO-1 in adipocytes produced from MSCs. This effect is associated with the suppression of Bach-1, a protein that negatively regulates HO-1 (Vanella et al., 2011; Burgess et al., 2012).

The overexpression of MSC-Exo with Nrf2/HO-1 has potential therapeutic applications for the treatment of various diseases, particularly diabetes. The exosomes have the ability to initiate the Nrf2/HO-1 pathway. MSC-Exo can alleviate dysfunction, inflammation, and ferroptosis in pulmonary microvascular endothelial cells induced by high glucose and lipopolysaccharide, which is particularly beneficial for diabetic patients who are more susceptible to infections and complications. By enhancing cell viability, migration, angiogenesis, and overall function, MSC-Exo acts as a mediator of the NRF2/HO-1 pathway, offering a novel strategy to alleviate diabetic complications and sepsis-induced injuries (Sun et al., 2023).

A prevalent complication of diabetes mellitus, diabetic retinopathy encompasses microangiopathy. It causes oxidative stress-related harm to retinal cells, especially retinal pigment epithelium cells. This results in a decline in vision and potential blindness, underscoring the imperative for a more profound comprehension of its mechanisms and the exploration of alternative therapeutic approaches. The MSC-sEV demonstrated a protective role in diabetic retinopathy (DR) by alleviating retinal oxidative stress and apoptosis. The PTEN/AKT/Nrf2 signaling pathway mediated the therapeutic effects. MSC-sEV facilitated the degradation of PTEN via MSC-sEV-delivered NEDD4, leading to enhanced AKT phosphorylation and increased Nrf2 expression. Consequently, this mechanism contributed to the prevention of DR progression. The findings highlight the crucial role of NRF2 and downstream effector HO-1 in MSC-sEV-mediated retinal protection, suggesting a potential therapeutic strategy for DR by targeting this signaling pathway (Sun et al., 2022a). MSC-EV therapy for diabetic retinopathy presents promising advantages, including a broad spectrum of biological activities that address multiple disease mechanisms, low immunogenicity, and a safer profile due to their non-integrating nature. However, it faces significant limitations such as challenges in delivery and retention within the retina, complex and costly production, and a lack of standardized regulatory frameworks. Compared to existing treatments like laser photocoagulation, VEGF inhibitors, and corticosteroids, which often target specific symptoms or disease aspects, MSC-EVs could potentially offer a more comprehensive approach by addressing inflammation, oxidative stress, and cell death. However, for MSC-EV therapy to be clinically feasible, improvements in the formulation and administration techniques are necessary to enhance delivery, efficacy, and the practicality of their use in clinical settings (Sun et al., 2022b).

Diabetic neuropathy is a common complication of prediabetes and diabetes. It is characterized by distal symmetric polyneuropathy, which causes sensory loss, discomfort, and significant morbidity. It affects at least 50% of people with diabetes. Present therapies prioritize glycemic control and pain mitigation, employing diverse strategies for managing type 1 and type 2 diabetes. Simultaneously, current research is investigating the intricate pathophysiology of neuropathy, with a focus on developing individualized therapy approaches and identifying modifiable risk factors to effectively address this debilitating condition (Callaghan et al., 2012; Rosenberger et al., 2020).

Shan et al. conducted a study to examine the impact of delivering exosomes produced from BMSC (bone marrow-derived mesenchymal stem cells) that overexpress sirtuin 1 (SIRT1), an antioxidant enzyme that relies on nicotinamide adenine dinucleotide (NAD+) and functions as a histone deacetylase, on diabetic rats with peripheral neuropathy. Administration of Exo-SIRT1 resulted in notable enhancements in neurological function, as demonstrated by increased nerve conduction velocity and decreased sensitivity to heat and mechanical stimuli, surpassing the benefits of Exo-control. Exo-SIRT1 activated the Nrf2/HO-1 signaling pathway, which resulted in the upregulation of TOMM20, a mitochondrial marker protein. This activation also led to a decrease in oxidative stress, an increase in the levels of antioxidants such as glutathione and superoxide dismutase, and an improvement in the mitochondrial membrane potential. These results highlight the therapeutic potential of Exo-SIRT1 in alleviating diabetic peripheral neuropathy through the modulation of Nrf2/HO-1-mediated antioxidant pathways and enhancement of mitochondrial function (Shan et al., 2022).

#### 3.9.2 Tooth

A recent study has highlighted the protective role of human ADSC-EVs in safeguarding xenogeneic bioengineered tooth roots (bio-roots) against oxidative damage. The human ADSC-EVs enhanced cellular viability, promoted migration, and boosted antioxidant capabilities. These protective effects are attributed to the activation of the PI3K/Akt pathway and Nrf2, leading to NRF2 nuclear translocation and subsequent upregulation of HO-1. Notably, inhibiting PI3K/Akt or silencing Nrf2 attenuates the antioxidant potential. In summary, the study underscores the significance of human ADSC-EVs as potential immune modulators and antioxidants for applications such as xenogeneic bio-root grafting and tissue regeneration, offering insights into mitigating oxidative stress-induced cellular damage (Fu et al., 2023; Luzuriaga et al., 2023).

#### 3.9.3 Toxins

Gao et al. conducted a study to examine the defensive properties of antioxidative extracellular vesicles (Antioxi-EVs) obtained from ADSC that overexpress Nrf2, in relation to lung injury caused by PM2.5. PM2.5 refers to fine airborne particles that have a size smaller than 2.5 μm. These particles are associated with respiratory and cardiovascular diseases because they contain sulfate, nitrate, heavy metals, and PAHs. These substances cause oxidative stress, production of reactive oxygen species (ROS), and inflammatory responses. However, the exact mechanisms and effective preventive treatments for these health issues are not yet fully understood. Exposure to PM2.5 resulted in oxidative stress, inflammation, and damage to the lungs. However, the use of Antioxi-EVs effectively reduced levels of reactive oxygen species (ROS), inflammatory cytokines, and lung injury. The Nrf2 pathway, together with the downstream effector HO-1, played a vital role in reducing oxidative stress. Antioxi-EVs exhibited enhanced antioxidative and anti-inflammatory properties in comparison to conventional EVs, indicating their ability to mitigate the toxicity caused by PM2.5 through Nrf2-mediated pathways (Gao et al., 2021).

## 4 Challenges and considerations

The journey of transforming research on EVs into therapeutic interventions presents numerous challenges and stringent regulatory requirements. The diversity within EV populations makes it difficult to standardize processes for their purification and analysis, which are critical for clinical use. Regulatory bodies such as the FDA demand detailed evidence of a therapeutic’s purity, effectiveness, safety, and strength, criteria that are particularly challenging to meet due to EV variability. Additionally, the technologies required for EV production, such as tangential flow filtration or immunocapture, are complex and costly, posing issues for large-scale production and ensuring the elimination of impurities. The stability and storage of EVs also need precise management to preserve their therapeutic properties over time. The lack of universally accepted markers for EV isolation and characterization further hinders the development of consistent protocols necessary for regulatory approval and clinical implementation. Addressing these obstacles is crucial for harnessing the full clinical potential of EVs (Cheng and Kalluri, 2023). EV heterogeneity is a central aspect complicating our understanding and utilization of EVs in regenerative medicine. These tiny membrane-bound particles exhibit diverse biophysical properties, cargo compositions, and functions, making their study and characterization challenging. Technical hurdles in separating and quantifying EV subtypes add layers of complexity, further compounded by the presence of non-vesicular co-isolates such as protein complexes and lipoproteins. Understanding the functional implications of EV heterogeneity is critical for defining therapeutically active EV subtypes and optimizing EV-based treatments for tissue regeneration. Addressing these challenges requires innovative approaches in EV isolation, characterization, and functional assessment, along with a deeper exploration of EV biogenesis pathways to unravel the origins of heterogeneity. Ultimately, deciphering the intricacies of EV heterogeneity holds the key to unlocking their full therapeutic potential in regenerative medicine (van de Wakker et al., 2023; Maličev and Jazbec, 2024). Scalability in EV production is a critical aspect for translating research findings into clinical applications. As the therapeutic potential of EVs becomes increasingly evident, the need for scalable manufacturing processes becomes more pronounced. The scalability of EV production hinges on several factors, including the selection of optimal cell sources, culture conditions, and downstream processing techniques. Efficient culture platforms, such as bioreactor systems, offer advantages in terms of scalability, enabling the production of larger quantities of EVs compared to traditional flask-based cultures. Moreover, the development of tiered cell banking systems allows for the expansion of cell banks from a single donor to produce multiple lots of EVs, enhancing scalability and reducing variability. Downstream processing techniques, such as filtration and chromatography, also play a crucial role in achieving scalable EV production by enabling efficient concentration and separation of EVs from culture media. By addressing scalability considerations early in the development process and implementing scalable manufacturing strategies, the EV field can better meet the growing demand for clinical-grade EV therapeutics (Adlerz et al., 2020).

## 5 Concluding remarks

**FIGURE 3:**
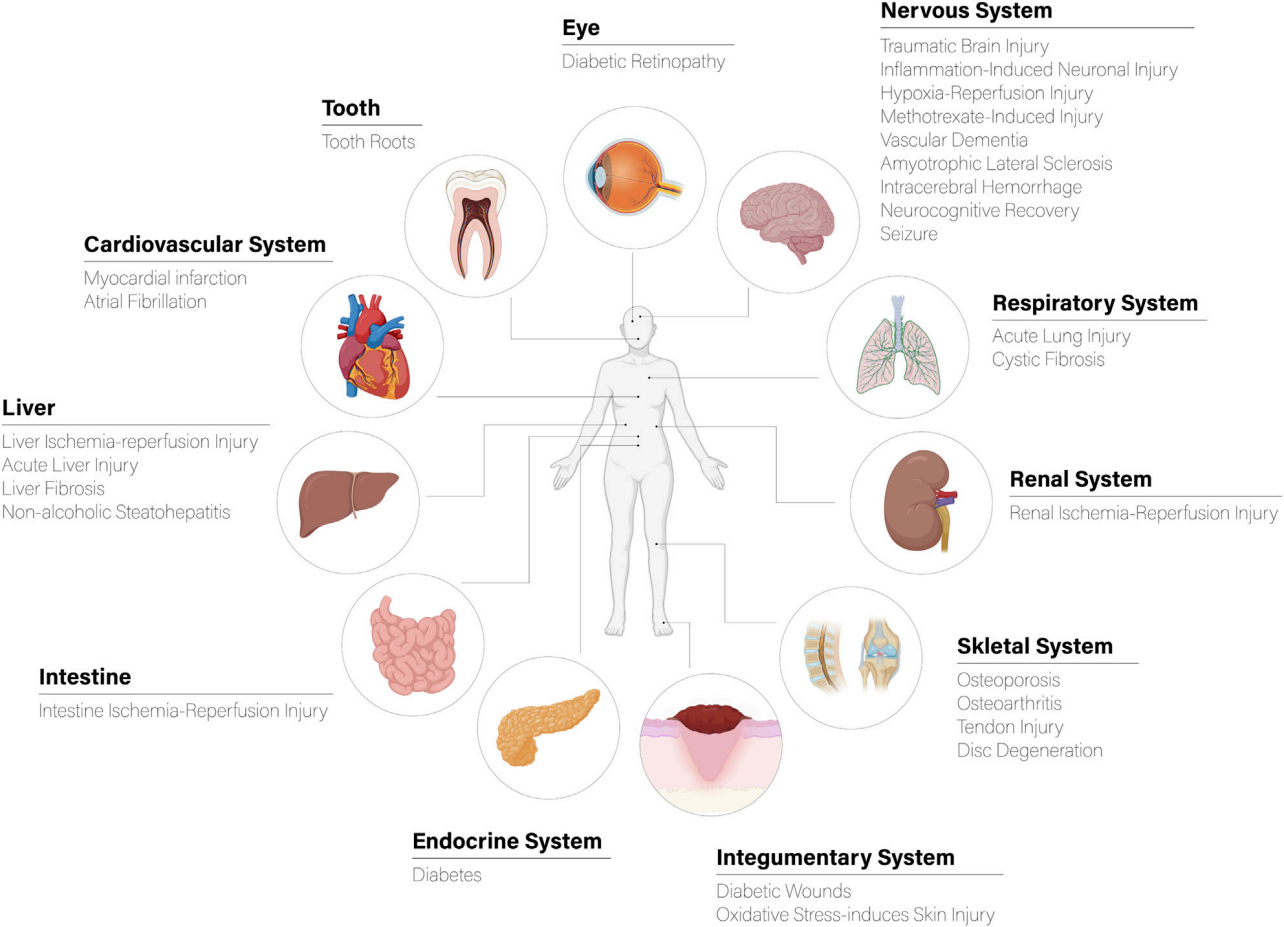
Human conditions affected by the MSC-EVs regulated Nrf2/HO-1 axis.

In conclusion, the extensive investigation into the therapeutic potential of MSC-EVs across various organ systems (Figure 3) presents a compelling narrative of their remarkable versatility and efficacy in addressing diverse medical conditions. From the integumentary system to the respiratory, digestive, cardiovascular, urinary, and skeletal systems, MSC-EVs consistently demonstrate regenerative properties mediated by activating the Nrf2/HO-1 pathway. The ability of MSC-EVs to accelerate wound healing in diabetic conditions, mitigate oxidative stress-induced skin injuries, and address acute lung injuries highlights their promise as innovative therapeutic agents. The findings from respiratory system studies, particularly in acute lung injury (ALI), underscore the significance of the Nrf2/HO-1 pathway in mediating anti-inflammatory and antioxidant effects. Furthermore, the administration route, such as inhalation of MSC-EVs, is critical in enhancing therapeutic efficacy. In the context of CF, lung MSC-EVs exhibit anti-inflammatory properties, suggesting their potential to mitigate hyper-inflammation in CF by targeting the PPARγ/NF-kB/HO-1 axis. The studies in liver ischemia-reperfusion injury, acute liver injury, and non-alcoholic steatohepatitis showcase innovative approaches involving HO-1-modified BMSCs and MSC-EVs, emphasizing their potential in overcoming challenges related to donor shortages and offering insights into the Nrf2/NQO-1 pathway in NASH. MSC-EVs demonstrate substantial promise in addressing myocardial infarction (MI) and atrial fibrillation (AF) in the cardiovascular system. Activating the Akt/Nrf2/HO-1 pathway and identifying crucial factors provide valuable insights into potential therapeutic strategies for these cardiac conditions. Moving to the urinary system, studies on renal ischemia-reperfusion injury highlight the regenerative effects of MSC-EVs through the Keap1-Nrf2 pathway, indicating their potential to protect against oxidative stress and inflammation. The superior protection demonstrated by melatonin-preconditioned MSC-Exo therapy further emphasizes the role of the antioxidant pathway HO-1 in renal injury. Lastly, investigations into the skeletal system, including osteoporosis, osteoarthritis, and disc degeneration, reveal the antioxidative and osteoprotective effects of MSC-EVs through Nrf2/HO-1-mediated pathways. These cumulative findings position MSC-EVs as promising candidates for future regenerative therapies, encouraging further exploration and clinical translation of these innovative interventions. The application prospects of MSC-EV technology in personalized and precision medicine are vast, offering a paradigm shift toward tailored therapeutic interventions. By harnessing disease-specific markers and advanced drug delivery technologies, MSC-EVs hold immense potential for achieving more targeted treatments. Incorporating disease-specific markers, such as genetic mutations, biomarkers, or imaging signatures, allows for the precise identification of pathological pathways and patient-specific disease characteristics. This personalized approach enables the customization of MSC-EV cargo to address the unique molecular signatures of individual patients, optimizing therapeutic efficacy while minimizing off-target effects. Moreover, coupling MSC-EVs with sophisticated drug delivery systems, such as nanoparticles or engineered biomaterials, enhances their precision by facilitating targeted delivery to specific tissues or cells within the body. These technologies enable the spatiotemporal control of therapeutic cargo release, ensuring optimal therapeutic concentrations at the disease site while minimizing systemic exposure. By integrating disease-specific markers with advanced drug delivery strategies, MSC-EV-based therapies have the potential to revolutionize personalized medicine by offering highly precise, tailored treatments that address the underlying mechanisms of disease with unprecedented accuracy and efficacy. The diversity in preparation and characterization methods of MSC-EVs poses a significant challenge to the repeatability and comparability of efficacy and mechanism studies in this field. To address this issue and promote the standardization and clinical translation of research results, the establishment of technical standards and quality control systems is urgently needed. These standards should encompass all aspects of MSC-EV isolation, purification, characterization, and storage to ensure consistency and reproducibility across different studies and laboratories. Key components of these standards could include guidelines for cell culture conditions, isolation protocols, characterization techniques (such as nanoparticle tracking analysis, electron microscopy, and protein analysis), and functional assays to assess MSC-EV potency and efficacy. Additionally, the implementation of robust quality control measures throughout the production process, including rigorous validation of reagents, equipment calibration, and documentation of procedures, is essential to ensure the reliability and safety of MSC-EV-based therapies. By establishing technical standards and quality control systems, the field can enhance the reliability and comparability of research outcomes, accelerate the translation of MSC-EV-based therapies into clinical practice, and ultimately maximize their potential to improve patient outcomes (Kolenc and Maličev, 2024; Tang et al., 2024).
